# Integration of co-culture conditions and 3D gelatin methacryloyl hydrogels to improve human-induced pluripotent stem cells-derived cardiomyocytes maturation

**DOI:** 10.3389/fbioe.2025.1576824

**Published:** 2025-07-14

**Authors:** Ilaria Gisone, Monica Boffito, Elisa Persiani, Roberta Pappalardo, Elisa Ceccherini, Andrea Alliaud, Manuela Cabiati, Rossella Laurano, Letizia Guiducci, Chiara Caselli, Rosetta Ragusa, Claudio Cassino, Chiara Ippolito, Silvia Del Ry, Susanna Sartori, Antonella Cecchettini, Salvador Fernández-Arroyo, Gianluca Ciardelli, Federico Vozzi

**Affiliations:** ^1^ Institute of Clinical Physiology IFC-CNR, Pisa, Italy; ^2^ Department of Mechanical and Aerospace Engineering, Politecnico di Torino, Turin, Italy; ^3^ Department of Science and Technological Innovation, Università del Piemonte Orientale “A. Avogadro”, Alessandria, Italy; ^4^ Department of Clinical and Experimental Medicine, University of Pisa, Pisa, Italy; ^5^ Centre for Omic Sciences (Joint Unit Eurecat Centre Tecnològic de Catalunya-Rovira i Virgili University), Reus, Spain

**Keywords:** hiPSCs differentiation, hiPSC-CMs culture, 3D model, hydrogels, gelatin methacryloyl

## Abstract

**Introduction:**

Human-induced pluripotent stem cell-derived cardiomyocytes (hiPSC-CMs) represent an excellent alternative to animals for *in vitro* cardiac studies. However, their immature fetal phenotype represents an important limit to consider. Approaches proposed to overcome this issue are based on better reproducing the *in vivo* native CMs microenvironment. In the present work, a biomimetic environment to enhance hiPSC-CMs maturation was developed by combining a 14-day co-culture of hiPSC-CMs and Human Coronary Artery Endothelial cells (HCAECs) in a 3D Gelatin Methacryloyl (GelMA) hydrogel system.

**Methods:**

Chemical characterization of custom-synthesized GelMA was performed through Attenuated Total Reflectance Fourier Transformed Infrared (ATR-FTIR) and proton Nuclear Magnetic Resonance (^1^H NMR) spectroscopies. GelMA degree of methacryloylation (DoM) was estimated through the ninhydrin colorimetric assay. Then, hydrogels were prepared by solubilizing GelMA in presence of phenyl-2,4,6-trimethyl-benzoyl phosphinate (LAP) as photoinitiator (0.05% w/v) and photo-rheological tests were carried out to investigate the photo-polymerization process (at 365 nm, 10 mW/cm^2^) and the mechanical properties of the resulting gels. Hydrogel swelling ratio was also monitored up to 5 days of incubation in aqueous medium at 37°C. The maturation phenotype was achieved by co-culturing hiPSC-CMs with HCAECs in the 3D model composed of GelMA with around 96% DoM, solubilized at 5% w/v concentration in cell culture medium, added with LAP and crosslinked by UV light (40 s). The expression of specific cardiac maturation markers was investigated through Real-Time PCR (RT-PCR). Omics analyses were carried out to compare terms of biological processes, cellular components, and molecular functions between the 3D model here presented and a classical 2D monoculture of hiPSC-CMs.

**Results:**

GelMA was successfully synthesized with two different DoMs (i.e., 30%–40% and 96%–97%) and used to prepare hydrogels at 5%, 7.5% and 10% w/v concentrations. Both GelMA DoM and hydrogel concentration appeared as tuning parameters of gel behavior in aqueous environment at 37°C and mechanical properties, with Young’s Modulus of photo-cured gels ranging between *ca.* 4 and 55 kPa. Within this plethora, photo-cured gels prepared from GelMA with *ca*. 96% DoM solubilized at 5% w/v concentration showed prolonged stability over time and E value (8.70 ± 0.12 kPa) similar to the native cardiac tissue and were thus selected to design bioengineered cardiac tissue models upon hiPSC-CMs and HCAECs loading. A direct comparison with the classical 2D monoculture of hiPSC-CMs highlighted the improved maturation profile achieved by hiPSC-CMs in the 3D GelMA system, as demonstrated by the higher expression of cardiac maturation markers (TNNT2, ACTN2, Myl2, MYH 7, CX43 and PPAR-α), in association with proteomics and transcriptomics data, that showed the modulation of specific biological pathways related to cardiac differentiation and contraction processes in the 3D system. A more in-depth investigation of cell health and function also suggested a higher viability and less suffering condition for cells co-cultured in the 3D hydrogel.

**Conclusion:**

Our results demonstrated that the 3D bioengineered model proposed here represents a good replica of the native cardiac tissue environment, improving the hiPSC-CMs maturation profile, thus opening the opportunity for its application in disease modeling and toxicological screening studies.

## 1 Introduction

Cardiovascular diseases are the leading cause of death globally, with an incidence of approximately 32% (in 2019) (Sharma et al., 2024). Considering the difficulties in working directly with human heart tissue and cells and the ethical concerns related to experimental animal models, there is a growing demand for new *in vitro* approaches to explore the microenvironment of the human heart better and, in turn, to perform patho-physiological and toxicological studies (Shi et al., 2020; Burnett et al., 2021; Krishna et al., 2021). Standard two-dimensional (2D) *in vitro* cell cultures have several advantages: easiness, reproducibility, low cost, simple analysis, and the possibility to control and manipulate the cellular environment (Notbohm et al., 2019; Wang et al., 2021). However, they also present some major drawbacks related to the failure in reproducing cell interactions occurring *in vivo* and the low yield in morphology, maturation, viability, gene/protein expression, and responsiveness to stimuli compared to the native tissue (Kapałczyńska et al., 2016; Tohyama et al., 2017; Ding et al., 2020). This forced their replacement with three-dimensional (3D) cultures, which, despite their complexity, provide more reliable systems to mimic the *in vivo* tissue environment, preserving cell phenotype and thus allowing a more realistic approach to exploring cell function and behavior (Gisone et al., 2022). Cardiomyocytes (CMs) are the prominent heart cell component (70%–80% of myocardial volume) responsible for its function (Notbohm et al., 2019). Due to the difficulties of working *in vitro* with adult CMs (non-dividing cells, limited lifespan and CMs death during cell isolation) (Woodcock and Matkovich, 2005; Talman and Kivelä, 2018; Notbohm et al., 2019; Onódi et al., 2022), many researchers turned to human-induced pluripotent stem cells (hiPSCs)-derived CMs (hiPSC-CMs), which provide an excellent alternative for safety assessment and disease modeling. hiPSCs can be expanded several times, maintaining their pluripotency. They can be differentiated into CMs using specific protocols with characteristics similar to those of primary human cardiomyocytes (Wang et al., 2021; Camman et al., 2022). Compared to adult ventricular CMs, hiPSC-CMs are immature, as confirmed by studies on their morphology, metabolism, beating contraction rate, myofibril alignment, and sarcomere organization (Denning et al., 2016; Ahmed et al., 2020; Camman et al., 2022). To better reproduce the complex *in vivo* microenvironment and improve hiPSC-CMs maturation, researchers proposed different approaches such as the co-culture of hiPSC-CMs with other cardiac cell types (endothelial cells (ECs) and/or cardiac fibroblasts (CFs)) (Giacomelli et al., 2020; Campostrini et al., 2021) that are in persistent paracrine communication with CMs (Sturtzel, 2017; Lother et al., 2018). Other researchers focused on 3D cultures that provide structural/architectural cues, where the cells are organized into a versatile and dynamic network that 2D culture cannot replicate (Lundy et al., 2013; Giacomelli et al., 2017; Andrysiak et al., 2021; Camman et al., 2022; Gisone et al., 2022). For this last aspect, biomaterials play a key role in defining 3D cell cultures, providing physical support for cell adhesion, proliferation, organization, and in case differentiation and adequate structural and mechanical stimuli driving cell behavior (Mathur et al., 2016; Caddeo et al., 2017). 3D frameworks can be obtained from natural or synthetic polymers or their combinations and processed as porous structures or bulk hydrogels. Hydrogels are three-dimensional, highly hydrated networks with a soft and rubbery consistency that allows them to recapitulate *in vitro* the texture of native soft tissues (Ho et al., 2022). Among hydrogel constituent materials, gelatin and its derivatives have been widely investigated in cardiac tissue engineering, as recently reviewed by Asl and colleagues (Asl et al., 2024). Specifically, hydrogels based on these materials hold great promise due to their compositional similarity to the native cardiac extracellular matrix (ECM) (Ju and Dixon, 1995), biocompatibility, low immunogenicity, biodegradability, viscoelastic properties, and low cost. Due to the upper critical gelation temperature (UCGT) behavior of gelatin aqueous solutions, hydrogel stabilization through chemical crosslinking is usually needed (Campiglio et al., 2019). The photo-polymerization of hydrogels based on gelatin derivatives bearing photo-curable moieties (e.g., gelatin methacryloyl (GelMA)) is a possible strategy for long-term hydrogel stabilization. GelMA hydrogels have been widely investigated in cardiac tissue engineering over the last decades. For instance, Zhang et al. have recently developed micropatterned GelMA hydrogels with varying geometrical features as promising substrates to enhance CM organization, maturation, and contraction (Zhang et al., 2024). In another work, to improve hydrogel similarity to the native cardiac ECM, GelMA was combined with a decellularized cardiac extracellular matrix (dECM), increasing cardiogenic gene expression and angiogenic potential of cardiac progenitor cells and endothelial cells, respectively (Bejleri et al., 2018). Conversely, Deidda and colleagues recently combined GelMA with xanthan gum to improve hydrogel printability (Deidda et al., 2024). The designed formulations showed enhanced printing accuracy and photo-cured gels exhibited stiffness similar to the native cardiac tissue and provided a proper environment for hiPSC differentiation towards the cardiac phenotype. Lastly, GelMA has also been combined with alginate, polypyrrole, and carboxyl-graphene to define a conductive hydrogel matrix supporting cell viability and proliferation, coupled with excellent mechanical performance, electrical conductivity, and hemocompatibility (Kaviani et al., 2024).

Based on these premises, the present work aims to combine the co-culture of cardiomyocytes with endothelial cells and a 3D GelMA hydrogel environment to achieve a better mimicry of cell life physiological conditions and improve CMs maturation. To this purpose, hiPSC-CMs and Human Coronary Artery Endothelial cells (HCAECs) were co-cultured in 2D and 3D *in vitro* models at two different ratios (90% hiPSC-CMs + 10% HCAECs and 80% hiPSC-CMs + 20% HCAECs) and compared with a hiPSC-CMs monoculture (100% hiPSC-CMs) to evaluate the impact of endothelial cells and the effects of the third dimensionality on hiPSC-CMs maturation process. Analysis of the expression of cardiac gene markers was performed to investigate the maturation phenotype of hiPSC-CMs in different conditions, with an additional characterization through the support of transcriptomics and proteomics, highlighting the most peculiar pathways involved in cardiac function and maturation. Additional biological assays investigated cell behavior in the 2D monoculture *versus* the defined 3D co-culture system. Our results highlight that the developed 3D model closely recapitulates the human heart tissue microenvironment, representing a reliable, high-throughput, and easy-to-assemble tool for patho-physiological studies and *in vitro* testing (e.g., drug screening and testing, cardiotoxicity assessment).

## 2 Materials and methods

### 2.1 2D culture of hiPSCs

Human induced pluripotent stem cells (hiPSCs, Gibco^®^ Episomal hiPSC Line) were cultured on Vitronectin (VTN-N, Gibco A14700)-coated flasks with Essential 8^®^ Flex basal medium (Gibco, A28583-01) + Supplement (Gibco, A28584-01) + 1% Penicillin/Streptomycin (P/S) (referred to as Complete Flex). Revita (Gibco A2644501-01) was added to Complete Flex (5 μL of Revita/mL of medium) just for the thawing and passaging of cells for 24 h. The medium was refreshed every 2 days, and at 70%–80% confluence, cells were gently expanded using Versene (Gibco, 15040-033) without destroying cell clusters. Cells seeding density was 20.000 cells/cm^2^.

### 2.2 hiPSCs differentiation in 2D culture

To start the differentiation protocol, clusters of hiPSCs (70%–80% of confluence) were rinsed twice with Dulbecco’s phosphate-buffered saline (DPBS) without calcium and magnesium (DBPS −/−), then detached and singularized with TrypLE (Gibco, A12177-01, diluted in Versene or DBPS −/−). Cells were seeded in VTN-N-coated flasks (seeding density of 20.000 cells/cm^2^), and the medium was replaced every 2 days until 50%–60% confluence. Then, the differentiation protocol started and continued for 12 days using the PSC Cardiomyocyte Differentiation Kit (Gibco, A29212-01). *Day 0*: Complete Flex was removed, and Medium A (Gibco, A29209-01) + 1% P/S was added. *Day 2*: Medium A was removed, and Medium B (Gibco, A29210-01) + 1% P/S was added. *Day 4*: Medium B was replaced with Cardiomyocyte Maintenance Medium (Gibco, A29208-01) and 1% P/S (referred to as Maintenance medium). The medium was refreshed on days 6, 8, and 10, till cells started to beat. On *day 12*, the cells were incubated with ROCK inhibitor (ROCKi, Y-27632) (Merk SCM075) (final concentration 10 μM in Maintenance medium) for 1 h at 37°C. Cells were then detached using TrypLE, and after centrifugation at 300xg for 5 min, the pellet was resuspended in Cryopreservation medium (Gibco, A26444-01) or Fetal Bovine Serum (FBS) supplemented with 10% dimethyl sulfoxide (DMSO) and stocked in liquid nitrogen.

### 2.3 HCAECs culture

Human Coronary Artery Endothelial Cells (HCAECs, PromoCell C-12221) were cultured and expanded with Endothelial Cell Basal Medium-2 (EBM™-2, Lonza CC-3156) + Supplement Pack (Lonza, CC-4176) + 15% FBS + 1% P/S.

### 2.4 hiPSC-CMs thawing and culture

Frozen differentiated cardiomyocytes (hiPSC-CMs) were quickly thawed in water at 37°C and, after centrifugation at 300xg for 5 min, the pellet was resuspended in Maintenance medium supplemented with 10% FBS and Revita (5 μL of Revita/mL of medium). Cells were seeded in VTN-N-coated flasks at a seeding density of approximately 100.000 cells/cm^2^. The next day, the medium was replaced with Maintenance medium without FBS and Revita, then refreshed every 2 days (with a post-thaw survival rate of approximately 40%). Beating contractions started approximately 5–7 days after thawing. At this point, cells were detached and used for the specific experiment.

### 2.5 Gelatin methacryloyl (GelMA)

#### 2.5.1 Synthesis protocol

GelMA synthesis was carried out by reacting gelatin (type A from porcine skin, Sigma Aldrich, Italy) and methacrylic anhydride (MA, Sigma Aldrich, Italy), which results in the grafting of methacrylate or methacrylamide groups to lysine and hydroxyl groups exposed along gelatin backbone, respectively (Ghosh et al., 2023) (See Supplementary Material Figure S1). The synthesis was performed according to the protocol described by Ghosh et al. (2023) and Yue et al. (2015), with slight modifications. Briefly, 10 g of gelatin were dissolved in phosphate buffered saline solution (PBS, pH 7.4, at 10% w/v) at 60°C and then, methacrylic anhydride (0.1 or 1 mL per g of gelatin, corresponding to a MA to gelatin -NH_2_ molar ratio of 40:1 and 400:1, respectively) was added dropwise while keeping the reaction mixture at 50°C. After 3 h, the synthesis was stopped by diluting the reaction mixture with PBS (at a 5:1 volume ratio with respect to the initial PBS volume used to solubilize gelatin). The resulting solution containing GelMA was then dialyzed (Spectrum dialysis tube with cut-off membrane 10–12 kDa) at room temperature (RT) for 1 week (complete dialysis medium refresh three times per day) and finally lyophilized (Martin Christ ALPHA 2-4 LSC Instrument, Germany).

Two different kinds of GelMA were synthesized, which differ in the degree of methacryloylation (DoM) according to the amount of MA used during the synthesis, in agreement with data reported by Gaglio et al. (2024). Hereafter, the synthesized GelMAs will be referred to with the acronyms GelMA_LOW and GelMA_HIGH, depending on the amount of MA added during the synthesis, namely 0.1 mL/g_gelatin_ or 1 mL/g_gelatin_, respectively.

#### 2.5.2 Chemical characterization

The synthesized GelMAs and gelatin (as control) were analyzed through Attenuated Total Reflectance Fourier Transformed Infrared (ATR-FTIR) and proton Nuclear Magnetic Resonance (^1^H NMR) spectroscopies. ATR-FTIR spectroscopy was performed using a Perkin-Elmer Spectrum 100 instrument equipped with an ATR accessory (UATR KRS5) with a diamond crystal. ATR-FTIR spectra were acquired at RT in the 4,000 to 600 cm^−1^ spectral range; each spectrum was obtained as a result of 16 scans with a resolution of 4 cm^−1^ and analyzed using the Perkin-Elmer Spectrum Software. ^1^H NMR characterization was performed on native gelatin (control) and the synthesized GelMAs to prove the successful functionalization of the gelatin backbone with methacrylate and/or methacrylamide groups. Spectra were obtained in deuterium oxide (D_2_O, 99.8%, Sigma Aldrich, Italy) through an Avance Neo Bruker spectrometer equipped with an 11.74 T superconductor magnet (500 MHz 1 H Larmor frequency) and a Bruker iProbe SmartProbe. All tests were performed at 25°C, and the spectra resulting from the average of 12 scans (10 s relaxation time) were referred to the D_2_O peak at 4.675 ppm. The registered spectra were further elaborated using MNova software (Mestrelab Research, S.L, Spain, www.mestrelab.com), utilizing the phenylalanine signal between 6.9 and 7.5 ppm to normalize all the spectra since this peak is proportional to polymer concentration.

Lastly, the Ninhydrin colorimetric assay, also commercially known as the Kaiser test, was used to indirectly estimate the methacryloylation degree (DoM) of the synthesized GelMAs. The Kaiser test kit used for these analyses was purchased from Sigma Aldrich, Italy, and the test was performed according to the supplier’s instructions. Briefly, GelMA and gelatin aqueous solutions (1% w/v in double demineralized water, ddH_2_O) were prepared, added with the Kaiser test reagents, mixed with a vortex, and heated at 120°C for 5 min in the dark. Then, they were diluted with an EtOH/ddH_2_O 60:40 v/v solution and analyzed using a Perkin Elmer (Waltham, MA, United States) Lambda 365 ultraviolet (UV)-Vis spectrophotometer, being the main absorption peak of the formed complexes between -NH_2_ groups and ninhydrin molecules at 570 nm. GelMA DoM was then defined using Equation 1

$$DoM=\frac{\left(A_{gelatin}\right)-\left(A_{\text{GelMA}}\right)}{\left(A_{\text{gelatin}}\right)}\cdot 100$$ (1)

where A_GelMA_ and A_gelatin_ represent the absorbance value measured at 570 nm for GelMA and gelatin samples, respectively.

The analyses were conducted in triplicate on gelatin, GelMA_LOW, and GelMA_HIGH, and the results were reported as mean ± standard deviation. Three different GelMA_LOW and GelMA_HIGH batches were synthesized and characterized to evaluate the repeatability of the synthesis process.

#### 2.5.3 Hydrogel preparation

Hydrogels were prepared at three different concentrations (5, 7.5, and 10% w/v) by solubilizing GelMA_HIGH or GelMA_LOW in PBS added with lithium phenyl-2,4,6-trimethyl-benzoyl phosphinate (LAP) at 0.05% w/v concentration. Briefly, the material was weighed and added with the required amount of LAP aqueous solution to achieve the targeted GelMA final concentration. Then, the formulation was mixed using a vortex and incubated at 37°C to favor GelMA solubilization. The developed hydrogel formulations are referred to with the acronym GelMA_X_Y%, where X identifies the kind of GelMA used for hydrogel preparation (with HIGH or LOW DoM), and Y refers to the polymer concentration within the formulation (5, 7.5, or 10% w/v).

#### 2.5.4 Hydrogel photo-rheological characterization

The capability of the prepared GelMA aqueous solutions to form crosslinked gels when cured with UV light was studied through photo-rheological analyses. Photo-rheological tests were carried out employing an Anton Paar Modular Compact Rheometer 302 (MCR 302, Anton Paar, Austria) equipped with a quartz lower plate for light irradiation, a 25 mm diameter parallel plate geometry, and a Peltier system for temperature control. Each sample was poured on the lower plate of the rheometer in the sol state (at 37°C), and a gap of 0.25 mm was set. Analyses were performed at 25°C, 0.1% strain (within the linear viscoelastic region according to Gaglio et al. (2024) and Rahali et al. (2017)), 1 Hz frequency, and normal force set at 0 N. A time sweep test was conducted for each sample after 10 minutes of equilibration at 25°C. The storage and loss moduli (G′ and G″, respectively) trends were initially registered for 1 min. The sample was then photo-cured by UV light (365 nm, 10 mW/cm^2^) for 90 s; finally, G′ and G″ trends were registered for an additional 60 s. The G′ and G″ values registered during the last 30 s of the time sweep test (i.e., after achievement of stable mechanical properties) were used to estimate the Young’s modulus (E) of photo-cured hydrogels according to Equation 2

$$E=2\cdot \sqrt{{G^{\prime }}^{2}+{G^{\prime \prime }}^{2}}\cdot \left(1+\vartheta \right)$$ (2)

where G′ and G″ identify the plateau storage and loss moduli values, and ϑ represents the Poisson’s ratio assumed to be 0.5 according to Yu et al. (2022).

Furthermore, the conducted photo-rheological characterization was employed to assess the hydrogel structural parameters (crosslinking density ρ, mol/m^3^, and mesh size ξ, nm) according to Equations 3, 4

$$\rho =\frac{G^{\prime }}{R\times T}$$ (3)

$$\xi ={\left(\rho \times N_{A}\right)}^{-1/3}$$ (4)

where G′ is the storage modulus value in the plateau region at the end of the photo-curing process, R, T and N_A_ represent the universal gas constant, the temperature and the Avogadro’s constant, respectively, according to Karvinen et al. (2019).

#### 2.5.5 Hydrogel behavior in contact with fluids

The behavior of photo-cured GelMA gels in contact with fluids was assessed in PBS at 37°C for up to 5 days of incubation. Specifically, GelMA gels were prepared at three different concentrations (5, 7.5, 10% w/v) by solubilizing GelMA_HIGH or GelMA_LOW in PBS solution containing LAP as photo-initiator (at 0.05% w/v concentration), in agreement with the protocol described in Paragraph 2.5.3. Then, 200 μL of GelMA_X_Y% solution were pipetted inside a ring-shaped mold with inner diameter of 1 cm, and photo-cured at 365 nm and 10 mW/cm^2^ for 60 s. Photo-cured GelMA_X_Y% disks were transferred in Bijou sample containers (17 mm diameter, 7 mL, Carlo Erba Reagents, Italy) and incubated in 500 μL of PBS, at 37°C (Memmert IF75, Schwabach, Germany). The PBS medium was completely refreshed every 3 days of incubation. Samples were collected at regular intervals (1, 3 and 5 days) by removing the residual buffer solution, and their wet mass was measured. Then, samples were freeze-dried and their dried mass was measured. After, the collected data were used to estimate the swelling ratio according to Equation 5

$$Swelling\;ratio=\frac{w_{wet\_t}-w_{dry\_t}}{w_{dry\_t}}$$ (5)

where w_wet_t_ identifies the measured wet weights of GelMA_X_Y% at the end of sample incubation in PBS at 37°C for 1, 3 and 5 days, respectively. Conversely, w_dry_t_ represents the weight of lyophilized samples after incubation in PBS for a defined time interval. In addition, the mesh size of GelMA gels was calculated from the gel weight measured after incubation in physiological-like conditions (i.e., pH 7.4, 37°C), the volume fraction and the GelMA hydrogel characteristic parameters, according to the methodology reported by Vigata et al. (2021). For a detailed description of the equations used, please refer to the Supplementary Information File.

### 2.6 hiPSC-CMs and HCAECs co-culture

#### 2.6.1 2D models

hiPSC-CMs and HCAECs co-cultures in a 2D microenvironment were obtained by mixing cells at different ratios: 90% hiPSC-CMs + 10% HCAECs and 80% hiPSC-CMs + 20% HCAECs. A parallel standard monoculture of 100% hiPSC-CMs was also carried out. 100.000 total cells/cm^2^ were seeded in Endothelial Cell Basal Medium-2 (EBM™-2, Lonza CC-3156) supplemented with 15% FBS, 1% P/S, and 0.5 ng/mL Vascular Endothelial Growth Factor (VEGF, Sigma, V7259) (referred to as complete EBM-2) + Revita (Revita was only kept for 24 h). Cells were maintained in culture for 14 days, changing the medium every 2 days. At the end of the 2 weeks, viability was investigated through the CellTiter-Blue^®^ assay. Cells were then collected and stored at −80°C until the cardiac genes’ expression analysis, proteomics and transcriptomics were performed.

#### 2.6.2 3D models

For 3D cardiac tissue model preparation, GelMA_HIGH was used upon sterilization through a standard ethylene oxide (EtO) method (Sterox Srl, Italy) and solubilized at 5% w/v concentration. In detail, EtO-sterilized GelMA_HIGH was solubilized in complete EBM-2 at 37°C, added with LAP, previously dissolved in complete EBM-2, and filtered using a PES filter with 0.22 μm pore size. The final solution containing 5% w/v GelMA_HIGH and 0.05% w/v LAP was used to resuspend the pellet of cells (100% hiPSC-CMs, 90% hiPSC-CMs + 10% HCAECs or 80% hiPSC-CMs + 20% HCAECs). 100 μL of cell-loaded GelMA_HIGH hydrogel (200.000 total cells) were seeded in 48 multiwell (MW) plates, and the photo-polymerization was performed through exposure to UV light (365 nm, 10 mW/cm^2^) for 40 s. Complete EBM-2 + Revita was added to hydrate cells inside the photo-cured gels. The following day, the medium was replaced with a same volume without Revita. The cells were co-cultured for 14 days in complete EBM-2, with refresh performed every 2 days. At the end of the 2 weeks of culture, viability was investigated through the CellTiter-Blue^®^ assay. Cells were then collected and stored at −80°C until cardiac genes’ expression analysis, proteomics and transcriptomics were performed.

### 2.7 Biological characterization

After identifying the co-culture condition in the 3D model that better enables hiPSC-CMs maturation, additional specific assays for biological characterization were performed on the selected system compared to a 2D monoculture of 100% hiPSC-CMs.

#### 2.7.1 CellTiter-blue^®^ assay

The CellTiter-Blue^®^ assay (Promega, G8081) is a fluorometric test that provides information about the viability of the cells by investigating their mitochondrial activity. To this aim, resazurin (blue solution) is added as a dye indicator that is converted into resorufin (pink solution) by living cells. CellTiter-Blue^®^ was diluted in the culture medium and then added to cell samples to perform the assay. After incubation for 2.5 h at 37°C, absorbance was read using a spectrofluorometer (resazurin and resorufin show maximum absorbance at 605 nm and 573 nm, respectively).

#### 2.7.2 Cytotoxicity detection assay

The Cytotoxicity Detection Kit (referred to as LDH assay) (Roche, 11644793001) is a colorimetric test to measure cell cytotoxicity by quantifying Lactate dehydrogenase (LDH), a cytoplasmatic enzyme released in the culture medium by damaged cells. Following the manufacturer’s instructions, the cell culture medium was mixed with a Reaction LDH solution. After incubation at RT for 30 min, the quantitative result was read using a spectrofluorometer (measuring the absorbance of the samples at 490 or 492 nm).

#### 2.7.3 ROS assay

The ROS-Glo™ H_2_O_2_ assay (Promega, G8821) is a luminescent test that measures the Reactive Oxygen Species (ROS) produced and released by cells by quantifying the hydrogen peroxide (H_2_O_2_) production. The H_2_O_2_ Substrate added to cell samples reacts with the H_2_O_2_ produced by the cells, resulting in a luciferin precursor that becomes luminescent upon reaction with a final detection reagent (containing an Ultra-Glo™ Recombinant Luciferase and D-Cysteine). Cells were exposed to the H_2_O_2_ Substrate for 5 h at 37°C; then, the medium was mixed with the detection solution following the manufacturer’s instructions. After incubation for 20 min at RT, luminescence was read using a luminometer. The luminescent signal is directly proportional to H_2_O_2_ concentrations.

#### 2.7.4 hsTnI

An automated platform based on the high sensitive immunoassay method (chemiluminescence microparticle immunoassay (CMIA)) was used to determine the levels of cardiac Troponin I (cTnI) in biological samples. The cell culture medium from each sample was prepared for analysis by dilution in physiological solution (manufacturer’s instructions were followed). Samples were then loaded into special tubes and placed into the instrument holder.

#### 2.7.5 Bio-molecular analysis: RNA extraction and Real-Time PCR experiments

Total RNA was extracted from samples at day 14 of cell culture by a dedicated kit (miRNeasy Mini Kit, Qiagen SpA, Milano, Italy) optimized to purify total RNA from small amounts of cells (<5∙10^5^). TrypLE was used to collect cells from 2D experiments. After centrifugation at 300xg for 5 min, the pellet was rinsed with DPBS −/− and centrifuged again. Regarding 3D samples, cell-loaded hydrogels were rinsed, collected into 2-mL Eppendorf tubes, and destroyed with a brief thermal shock (15 min in liquid nitrogen) followed by mechanical destruction with Tissue Lyser (Qiagen S.p.A.) at 25 Hz for 2 min.

Total RNA was extracted by acid guanidinium thiocyanate-phenol-chloroform (Qiazol) following the manufacturer’s instructions (Qiagen SpA, Milano, Italy); after re-suspension and lysis of the cells, ethanol was added to the flow-through to provide appropriate binding conditions for RNA. Then, the samples were selectively bound to a silica-based membrane (RNeasy MinElute spin column) and centrifuged at 12,000 rpm for 30 s on a microspin centrifuge. A specific high-salt buffer system allowed RNA to bind to the RNeasy silica membrane, and contaminants were washed out. High-quality RNA was eluted in 30 μL of RNAse-free water without additional DNase digestion. The total RNA sample concentration was determined by measuring the absorbance at 260 (A260) and 280 (A280) nm (NanoDrop Thermofisher, Waltham, MA, United States) and calculated using the Beer-Lambert law (expected A260/A280 ratio value between 1.8 and 2.1). The RNA samples were stored at −80°C for use in gene expression studies.

First-strand cDNA was synthesized by IScript cDNA Synthesis Kit (Bio-Rad Laboratories Inc., Hercules, CA, United States), which uses the Moloney Murine Leukaemia Virus (M-MuLV) reverse transcriptase, optimized for reliable cDNA synthesis over 1 μg of total RNA as template. The reverse transcriptase reaction sequence consisted of incubation at 25°C for 5 min, followed by three different cycles at 42°C for 30 min and 45°C–48°C for 10 min to separate the strands better. The reverse transcriptase enzyme was inactivated by heating to 85°C for 5 min. The cDNA samples were diluted appropriately and stored at +4°C.

Gene expression was determined by Real-Time PCR performed in duplicate on the Bio-Rad C1000 TM thermal cycler (CFX-96 Real-Time PCR detection systems, Bio-Rad). The third-generation fluorophore EvaGreen was used to monitor cDNA amplification (SsoFAST EvaGreen Supermix, Bio-Rad). The primers for reference and target genes were accurately designed using dedicated software such as oligo architect^®^ (Sigma Aldrich-Merck), which refers to the nucleotide sequences contained in the GenBank database of the NCBI. Specific primers were designed for exon-exon junctions whenever possible to avoid amplifying genomic DNA. The amplification protocol started at 98°C for 30 s, followed by 40 cycles at 95°C for 5 s. Amplifiers were systematically checked by melting curve analysis to assess product specificity. Melting curves were generated from 65°C to 95°C with increments of 0.5°C/cycle. All the annealing temperatures were 58°C–60°C with a reaction efficiency of about 95%–105%.

The MIQE Guidelines (Bustin et al., 2009) for a correct and reproducible Real-Time PCR experiment were followed. The GeNorm technology integrated into Bio-Rad’s CFX96 manager software (CFX-96 Real-Time PCR detection systems, Bio-Rad Laboratories Inc., Hercules, CA) was used to establish the most stably expressed genes, as described by Vandesompele et al. (2002). The mRNA expression data were normalized by the geometric mean of the three most stably expressed genes (RPL13a, RPS4X, and PPIA, M value <1.0) evaluated among approximately 10 reference genes, and relative quantification was performed by the ∆∆Ct method. Raw data were then normalized for the viability value (CellTiter-Blue assay). The primers are listed in the Supplementary Information File (see Supplementary Material Table S1).

#### 2.7.6 Live-dead assay

The Live-Dead cell viability assay (Sigma, CBA415) is a three-color assay that measures cell viability based on plasma membrane integrity and esterase activity, due to the Calcein-AM and Propidium iodide dyes, together with Hoechst. The non-fluorescent and highly lipophilic Calcein-AM dye is converted into a green, fluorescent dye (Calcein) by esterase of viable cells, thus it stains only live cells, while the red dye Propidium iodide cannot pass through the cell membrane, thus it can stain only the nucleus of dead cells. Hoechst dye 33342 is used to stain all nuclei (blue stain). All dyes were diluted in a culture medium solution and PBS (1:1 ratio) following the manufacturer’s instructions and then added to cell samples. After incubation for 1 h (for 3D samples) at 37°C, a laser scanner confocal microscopy Leica TCS SP8 was used to capture images of cells and perform a z-stacked picture, using 490, 535 and 361 nm wavelengths for Calcein AM, Propidium iodide and Hoechst, respectively.

#### 2.7.7 Immunofluorescence microscopy

Immunofluorescence analysis was performed to confirm the effective differentiation of hiPSCs into hiPSC-CMs. Both hiPSCs and hiPSC-CMs were seeded on Willco chambers and cultured for 14 days. Cells were fixed with 2% paraformaldehyde (PFA) at 4°C for 15 min, rinsed twice with PBS −/− and stained using the immunocytochemistry kit (Thermo Fisher, A25973), following the manufacturer’s instructions. Fixed cells were permeabilized with 1% saponin in DPBS for 15 min at RT and blocked with 3% bovine serum albumin (BSA) in DPBS at RT for 30 min. Samples were incubated overnight (O/N) at 4°C with primary antibodies (mouse anti-TNNT2 or anti-ACTN2), diluted to 1:1,000 in blocking solution. Cells were then washed twice with PBS, and they were incubated for 1 h at RT with secondary antibodies (Alexa Fluor^®^ 488 green: donkey anti-mouse anti-TNNT2 or anti-ACTN2), diluted in blocking solution to yield a 1:250 final dilution. Cells were washed twice with a washing buffer and once with sterile water. The slides were mounted using the Fluoroshield™ with DAPI (4′,6-diamidino-2-phenylindole) (Sigma, F6057). The laser scanner Leica TCS SP8 confocal microscope was used with 405 nm and 488 nm excitation lasers and the zeta stuck function to capture images of cells.

### 2.8 Transcriptomics

#### 2.8.1 Extraction of RNA and quality assessment

RNA was extracted from cell pellets and cells embedded in hydrogel as described above. The extracted RNA was quantified using a qubit fluorometer. The quality and integrity (RIN) of the RNA samples were evaluated using the Agilent TapeStation system and the Agilent RNA ScreenTape Assay, respectively.

#### 2.8.2 Creation and quantification of sequencing libraries

For library preparation, the Illumina Stranded mRNA Prep kit (20040534, Illumina) was utilized following the manufacturer’s instructions. A total of 500 ng of initial RNA was used. This process involves fragmentation of RNA, retro-transcription, and modification to incorporate adapter sequences and unique short sequences called “indexes,” which enable sample differentiation during subsequent data analysis. The resulting DNA libraries, containing retrotranscribed and modified RNA fragments, were quantified using a microfluidic electrophoresis on the Agilent TapeStation instrument and the Agilent DNA High Sensitivity ScreenTape kit. The length and concentration of each library were determined based on these measurements. Equimolar pools were created by combining the libraries at a final concentration of 750 pM.

#### 2.8.3 Massive sequencing on the illumina platform

The library pools were sequenced using the Illumina NextSeq2000 instrument, generating approximately 30 million paired-end reads of 76 base pairs (2 × 76 bp) per sample.

#### 2.8.4 Bioinformatic analysis

The data analysis pipeline involved mapping the reads to a reference genome using HISAT2 (version 2.2.1), then annotating and quantifying the aligned reads using StringTie (version 2.1.4).

### 2.9 Proteomics

#### 2.9.1 Protein extraction and quantification for cell samples

Cell lysis and protein extraction from cell pellets or cells embedded in GelMA gels were performed according to the RIPA buffer protocol (Fisher Scientific). First, proteins were extracted by adding 0.5 mL of RIPA buffer and homogenizing it with an ultrasonic probe (3 cycles, 20% amplitude, 10 s/cycle) for 1 min. Afterward, homogenized extracts were incubated under stirring for 15 min at 4°C and centrifuged at 14,000 rpm for 15 min. The supernatants were collected with 10% trichloroacetic acid/acetone for protein precipitation. Then, protein pellets were resuspended in 6 M urea/50 mM ammonium bicarbonate for digestion.

#### 2.9.2 Protein digestion and peptide 11-plex TMT labeling

20 µg of total protein (quantified by Bradford’s method) were reduced with 4 mM 1.4-dithiothreitol for 1 h at 37°C and alquilated with 8 mM iodoacetamide for 30 min at 25°C in the dark. Afterward, samples were overnight digested (pH 8.0, 37°C) with sequencing-grade Trypsin/Lys-C Protease Mix (ThermoFisher Scientific, CA, United States) at enzyme:protein ratio of 1:50. Digestion was quenched by acidification with 1% v/v formic acid and peptides were desalted on Oasis HLB SPE column (Waters, Massachusetts, United States) before TMT 11-plex labeling (ThermoFisher Scientific, CA, United States) following manufacturer’s instructions.

A pool of all samples was labeled with a TMT-126 tag and included in each TMT batch to calculate relative protein ratios. The different TMT 11-plex batches were desalted on Oasis HLB SPE columns before the nanoLC-MS analysis.

#### 2.9.3 nanoLC-(Orbitrap)MS/MS analysis

Labeled and multiplexed peptides were loaded on an Acclaim PepMap nano-trap column (100 μm I.D.; 2 cm length; 5 μm particle diameter, ThermoFisher Scientific, CA, United States) and separated onto a C18 reversed phase (RP) nano-column (75 μm I.D.; 15 cm length; 3 μm particle diameter, Nikkyo Technos Co. LTD, Japan) on an EASY-II nanoLC from ThermoFisher. The chromatographic separation was performed with a 180-min gradient using Milli-Q water (0.1% formic acid) and acetonitrile (0.1% formic acid) as mobile phase at a 300 nL/min flow rate.

Mass spectrometry (MS) analyses were performed on an LTQ-Orbitrap Velos Pro from ThermoFisher by an enhanced FT-resolution MS spectrum (R = 30,000 FHMW) followed by a data-dependent FT-MS/MS acquisition (R = 15,000 FHMW, 40% NCE HCD) from the most intense ten parent ions with a charge state rejection of one and dynamic exclusion of 0.5 min.

#### 2.9.4 Protein identification/quantification

Protein identification/quantification was performed on Proteome Discoverer software v.1.4.0.288 (ThermoFisher Scientific, CA, United States) by Multidimensional Protein Identification Technology (MudPIT), combining the raw data files obtained from each sample fraction.

For protein identification, all MS and MS/MS spectra were analyzed using the Mascot search engine (v.2.5). The workflow was set up using two different Mascot nodes combining the *Homo Sapiens* database (74,449 entries) and contaminants database (247 entries) both searches assuming trypsin digestion. Two missed cleavages were allowed, and an error of 0.02 Da for an FT-MS/MS fragmentation mass and 10.0 ppm for an FT-MS parent ion mass were allowed. TMT-10plex was set as quantification modification, methionine oxidation, and N-termini acetylation were set as dynamic modifications, whereas carbamidomethylation of cysteine was set as static modification. The Percolator calculated the false discovery rate (FDR) and protein probabilities. The protein network visualization was obtained using STRING.

### 2.10 Statistical analysis

Results were expressed as mean ± SEM, if not differently expressed. Data were evaluated for their normal distribution. Parametric and non-parametric t-tests were done for comparison in pair, while 2-way ANOVA analysis was used for multiple comparisons. The means were statistically compared using Student’s independent t-test. Each experiment was performed at least in duplicate. Regarding molecular investigations, the mRNA expression data were normalized by the geometric mean of the most stably expressed genes (RPL13a, RPS4X, and PPIA), and the relative quantification was performed by the ∆∆Ct method. When the expression values were not normally distributed, the data logarithmic transformation was used for statistical analysis. The Fisher’s test was used after ANOVA. Real-Time PCR experiments were performed in duplicate. *p* < 0.05 was considered significant. The statistical analysis of the results was carried out through the Stat-View 5.0.1 software released for Windows Statistical (1992-98, SAS Institute Inc., SAS Campus Drive, Cary, NC, United States) and GraphPad Prism 9.0.1 software.

## 3 Results

### 3.1 GelMA characterization

#### 3.1.1 GelMA chemical characterization

**FIGURE 1:**
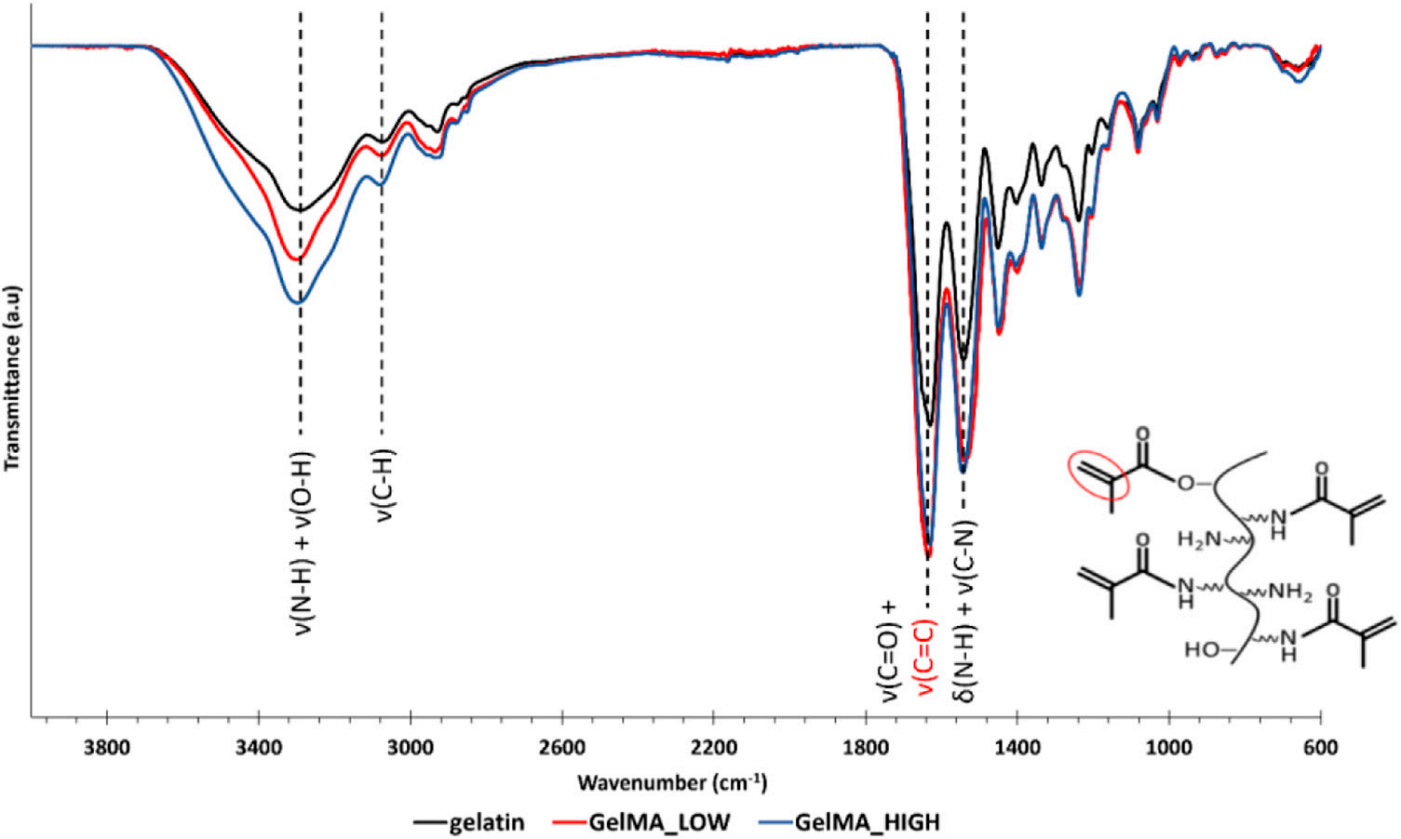
ATR-FTIR spectra of gelatin (black), GelMA_LOW (red), and GelMA_HIGH (blue). Dashed lines identify the characteristic bands of gelatin and GelMA.

**FIGURE 2:**
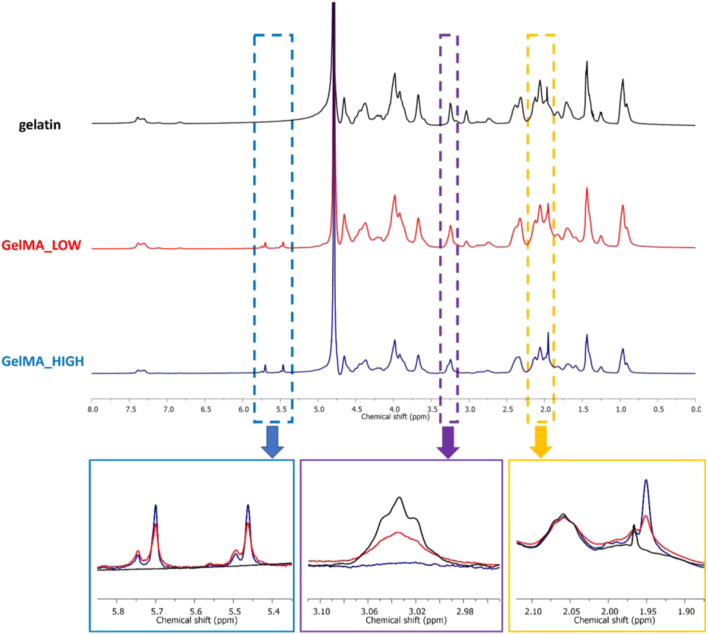
^1^H NMR spectra of gelatin (black), GelMA_LOW (red) and GelMA_HIGH (blue). The blue rectangle underlines the region of the spectra containing the signals due to the acrylic protons (CH_2_ = C(CH_3_)CONH) of methacrylamide groups; the purple rectangle highlights the chemical shift range where the free lysine methylene signal (NH_2_CH_2_CH_2_CH_2_CH_2_-) appears; and the orange rectangle underlines the region of the spectra with the signal at 1.85 ppm due to the methyl protons (CH_2_ = C(CH_3_)CO-) of methacryloyl groups.

The as-synthesized GelMAs was first spectroscopically characterized by ATR-FTIR and ^1^H NMR spectroscopies to prove the success of the synthesis process. Figure 1 reports the ATR-FTIR spectra of GelMA_LOW, GelMA_HIGH, and virgin gelatin as control. Spectra comparison immediately showed that the characteristic peaks of methacryloyl groups and unmodified gelatin overlapped. For instance, the stretching vibration of C=C bonds in the pendant methacryloyl groups overlapped with the characteristic amide I band of gelatin at around 1,627 cm^-1^. However, the increased intensity of the signals at 1,627 cm^-1^ (stretching of C=O and C=C bonds), 3,000 cm^-1^ (stretching vibrations of C-H), and 1,500 cm^-1^ (C-N stretching vibration) indirectly proved the effective grafting of methacryloyl moieties to the gelatin backbone. Conversely, a direct proof of the effective synthesis of GelMA with two different DoM values was provided by ^1^H NMR spectroscopy. Figure 2 compares the registered ^1^H NMR spectra for gelatin, GelMA_LOW, and GelMA_HIGH. The free lysine methylene signal (NH_2_CH_2_CH_2_CH_2_CH_2_-) at *ca.* 3.0 ppm decreased in GelMA_HIGH and GelMA_LOW samples with respect to native gelatin because of the reaction of the amino groups in its lateral chains with methacrylic anhydride, leading to the formation of amidic bonds and the grafting of methacrylamide groups. New peaks also appeared in GelMA_HIGH and GelMA_LOW spectra proving the success of the methacryloylation reaction: the two peaks in the region between 5.8 and 5.4 ppm can be ascribed to the acrylic protons (CH_2_=C(CH_3_)CONH) of methacrylamide groups, while the signal at 1.95 ppm is due to the methyl protons (CH_2_=C(CH_3_)CO-) of methacryloyl moieties. At high MA concentrations, the literature also reports the occurrence of an additional methacryloylation reaction between the hydroxyl groups of hydroxyproline and methacrylic anhydride molecules, which leads to the grafting of methacrylate groups through ester bonds (Zhu et al., 2019). However, this additional reaction turned out to be almost negligible in the synthesis of GelMA_LOW and GelMA_HIGH since no peaks at 6.1 and 5.7 ppm (acrylic protons (CH_2_ = C(CH_3_)COO-) of methacrylate groups) were present in both GelMA_LOW and GelMA_HIGH spectra.

**FIGURE 3:**
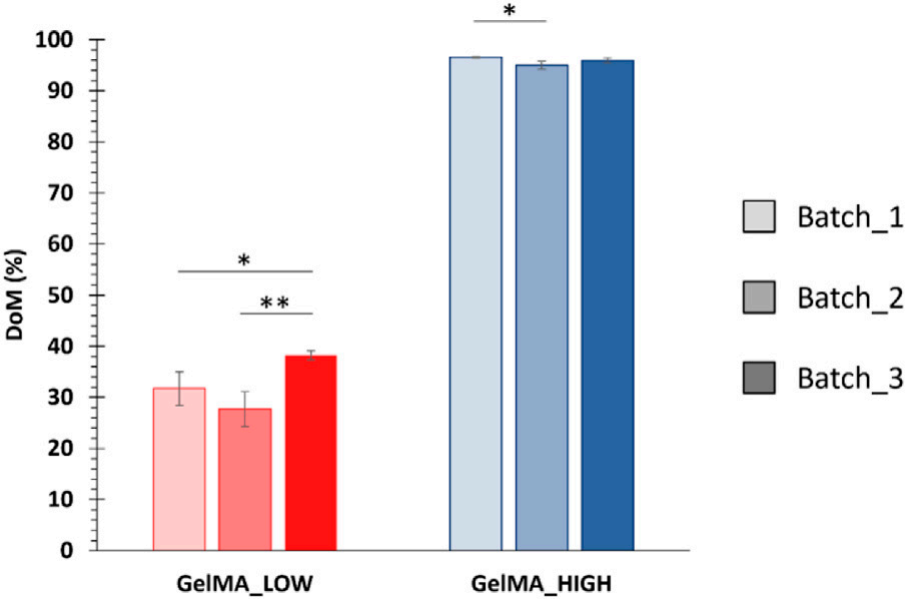
DoM values estimated through the Ninhydrin assay for different GelMA batches (i.e., 3 batches for both GelMA_LOW and GelMA_HIGH) (means compared using Student’s independent t-test. *p from 0.01 to 0.05; **p from 0.001 to 0.01, n = 3, data reported as mean ± SD).

Lastly, the degree of methacryloylation of GelMA_LOW and GelMA_HIGH was estimated using the Ninhydrin assay by correlating the amount of amino groups exposed along the native gelatin backbone to the residual amino groups present along GelMA_LOW and GelMA_HIGH chains. The DoM values estimated for three independently performed GelMA_LOW and GelMA_HIGH syntheses are summarized in Figure 3. The DoM of GelMA_LOW and GelMA_HIGH varied between 30%–40% and 96%–97%, respectively.

#### 3.1.2 Photo-rheological characterization of GelMA hydrogels

**FIGURE 4:**
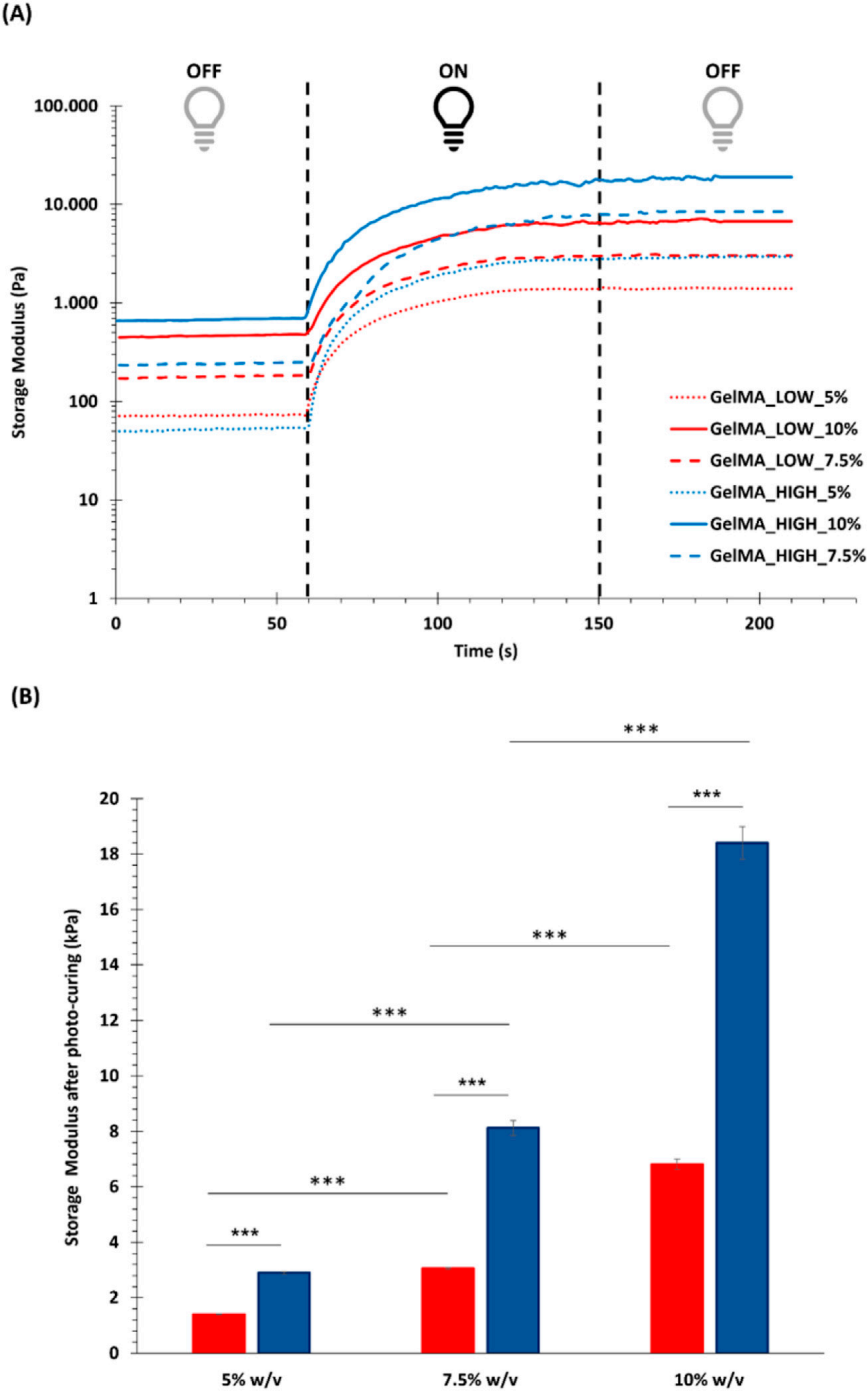
Results of the photo-rheological characterization performed on GelMA formulations. **(A)** Trend of storage modulus (G′) as a function of time, as registered during photo-rheological tests (60s light OFF + 90s light ON + 60s light OFF) carried out on GelMA_LOW_5%, GelMA_LOW_7.5%, GelMA_LOW_10%, GelMA_HIGH_5%, GelMA_HIGH_7.5%, and GelMA_HIGH_10% (red and blue tones for GelMA_LOW and GelMA_HIGH formulations, respectively); **(B)** Value of storage modulus registered at the end of the photo-rheological time sweep tests for all the investigated GelMA formulations (red and blue for GelMA_LOW and GelMA_HIGH formulations, respectively) (means were statistically compared using Student’s independent t-test. ***p from 0.0001 to 0.001, n = 3, data reported as mean ± SD).

**FIGURE 5:**
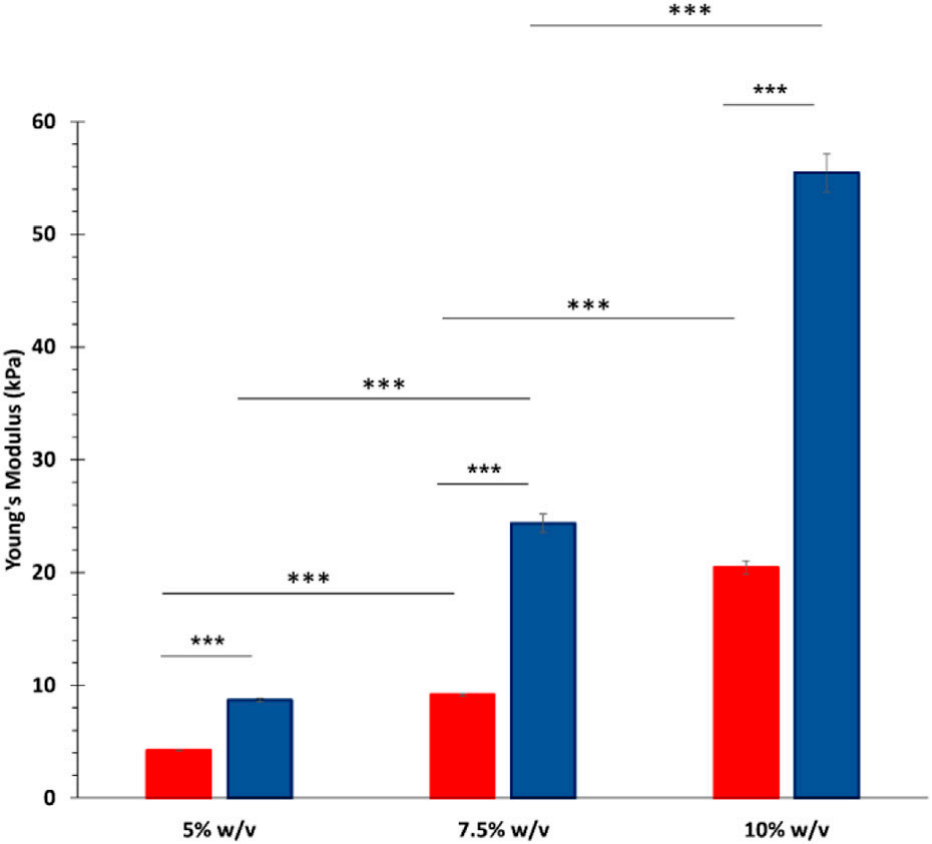
Young’s modulus of photo-crosslinked gels calculated based on G′ and G″ values, as measured upon photo-curing of GelMA-HIGH and GelMA-LOW aqueous solutions. In the figure, red and blue colors are used for GelMA_LOW and GelMA_HIGH formulations, respectively. (means were statistically compared using Student’s independent t-test. ***p from 0.0001 to 0.001, n = 3, data reported as mean ± SD).

Photo-rheological tests were conducted to assess the capability of GelMA aqueous solutions to form photo-crosslinked gels and study the kinetics of the photo-polymerization process initiated by UV light irradiation. Figure 4A reports the registered trends of G′ as a function of time during photo-rheological characterization. As expected, the G′ value initially remained constant as no physico-chemical transition occurred within the samples (the light source was turned off). Then, with the beginning of the light irradiation step (from 60 to 150 s), the G′ value sharply increased due to the formation of chemical crosslinks among the polymer chains. Starting from around 100 s (i.e., after 40 s of light irradiation), the G′ trend became progressively constant again, suggesting the completion of the chemical crosslinking reaction (Figure 4A). Figure 4B summarizes the storage modulus values registered at the end of the photo-rheological time sweep tests for all the designed GelMA formulations. Both GelMA DoM and concentration within the hydrogels turned out to affect the photo-curing process and its outcomes. Concerning hydrogel concentration, by increasing the concentration of GelMA aqueous solution from 5% to 10% w/v, both the pre- and post-curing storage modulus values increased. In detail, irrespective of GelMA DoM, with increasing hydrogel concentration from 5% w/v to 7.5% w/v, the registered G′ value at the end of the irradiation interval increased *ca.* 2-3 folds (significant difference); a similar fold change increase (significant difference) was also observed with further increasing GelMA concentration from 7.5% w/v to 10% w/v. Hence, a 2.5% w/v increase in GelMA concentration resulted in the introduction of a number of methacryloyl functionalities within the samples that significantly affected the final mechanical properties achieved upon the photo-curing step. Similarly, the degree of methacryloylation also significantly influenced the mechanical performances of photo-irradiated samples. As highlighted in Figure 4B, the difference in the degree of methacryloylation between GelMA_LOW and GelMA_HIGH significantly affected the final G′ value achieved at the end of the photo-curing step. Indeed, being GelMA concentration the same, an approx. 2-3 fold increase in the G′ value was measured after the photo-curing step with increasing GelMA DoM from 30%–40% to 96%–97%. Lastly, the G′ and G″ values registered during the last 30 s of the time sweep test were used to estimate the Young’s modulus, the crosslinking density (ρ) and the mesh size (ξ) of photo-cured gels according to Equations 2–4, respectively. The estimated E values are summarized in Figure 5 and followed a similar trend to the one observed for the measured G′ values at the end of the photo-polymerization reaction. Again, GelMA DoM and concentration were pivotal in determining gel stiffness. Irrespective of GelMA DoM, the Young’s modulus significantly increased with increasing hydrogel polymeric concentration: a 2.5% w/v increase in hydrogel concentration resulted in around a 2-3 fold increase in the gel E value. For instance, for GelMA_LOW, the E value of photo-crosslinked gels was estimated to be 4.20 ± 0.05 kPa and 9.17 ± 0.10 kPa at 5% and 7.5% w/v polymer concentration (significant difference), respectively. Similarly, irrespective of hydrogel polymeric concentration, the estimated Young’s modulus of photo-polymerized gels increased with increasing GelMA DoM due to the increased number of methacryloyl moieties available for forming chemical bonds among the polymer chains. For instance, at 5% w/v concentration, GelMA_LOW- and GelMA_HIGH-based gels exhibited Young’s modulus values of 4.20 ± 0.05 kPa and 8.70 ± 0.12 kPa, respectively. The estimated values of crosslinking density (ρ) and the mesh size (ξ) for GelMA_LOW and GelMA_HIGH gels are collected in Table 1. As expected, irrespective of GelMA DOM, ρ increased with increasing hydrogel concentration, in agreement with the increasing number of methacryloyl functionalities present within the samples and available for covalent bond formation during light irradiation. Accordingly, ξ decreased with increasing gel crosslinking density. Analogously, hydrogel concentration being the same, GelMA_HIGH-based samples showed higher crosslinking density and lower mesh size compared to GelMA_LOW-based gels. These structural changes are perfectly in agreement with the trend observed for the Young’s Modulus values. Interestingly, to achieve similar structural characteristics, GelMA_LOW and GelMA_HIGH gels should be formulated with a concentration difference of 2.5% w/v, as highlighted by comparing GelMA_LOW_7.5% and GelMA_HIGH_5% that also exhibited not statistically different E values (i.e., 9.17 ± 0.10 kPa and 8.70 ± 0.12 kPa, respectively).

**TABLE 1:** Crosslinking density (ρ) and mesh size (ξ), as estimated starting from rheological data for GelMA_LOW and GelMA_HIGH photo-crosslinked gels.

GelMA	Concentration	Crosslinking density (ρ)	Mesh size (ξ, nm)
GelMA_LOW	5% w/v	0.6	14.3
	7.5% w/v	1.2	11.0
	10% w/v	2.7	8.5
GelMA_HIGH	5% w/v	1.2	11.2
	7.5% w/v	3.3	8.0
	10% w/v	7.4	6.0

#### 3.1.3 GelMA gel behavior in contact with fluids

**FIGURE 6:**
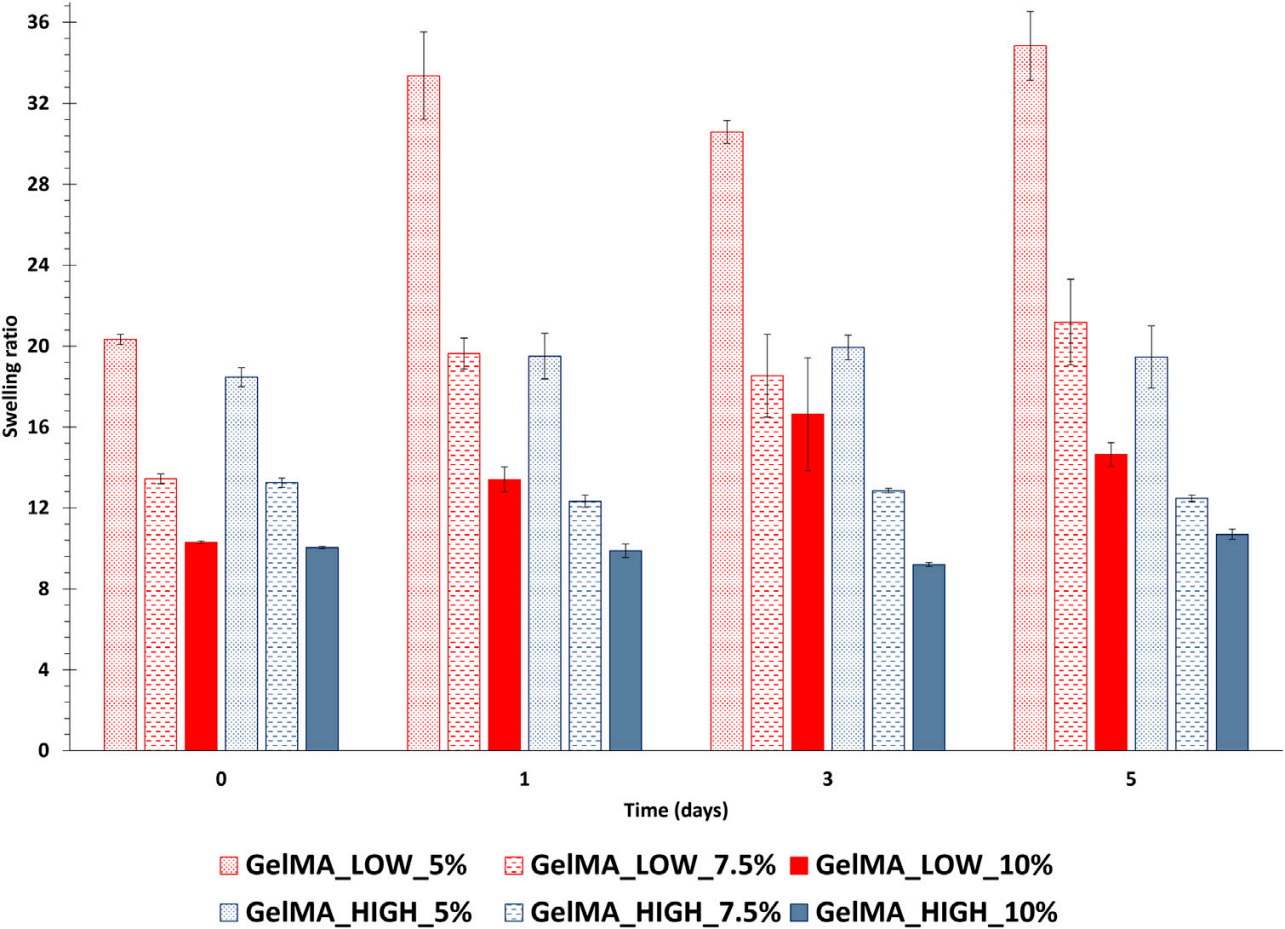
Swelling ratio of photo-crosslinked GelMA_LOW and GelMA_HIGH gels after 1, 3 and 5 days of incubation in PBS at 37°C. The initial gel swelling ratio (i.e., relaxed swelling ratio, Q_mr_), is also reported (data at 0 days incubation time). In the figure, red and blue tones are used for GelMA_LOW and GelMA_HIGH formulations, respectively (dotted, dashed and plain colors identify gels prepared at 5, 7.5 and 10% w/v concentration, respectively). (data were statistically compared using ANOVA followed by Bonferroni post-test. ***p from 0.0001 to 0.001, **p from 0.001 to 0.01, *p from 0.01 to 0.05, ns *p* ≥ 0.05, n = 3, data reported as mean ± SD). (GelMA_LOW_5: 0 days *vs.* 1 day ***; 1 day *vs.* 3 days *; 3 days *vs.* 5 days ***; GelMA_LOW_7.5: 0 days *vs.* 1 day ***; 1 day *vs.* 3 days ns; 3 days *vs.* 5 days **; GelMA_LOW_10: 0 days *vs.* 1 day **; 1 day *vs.* 3 days **; 3 days *vs.* 5 days ns; GelMA_HIGH_5: 0 days *vs.* 1 day ns; 1 day *vs.* 3 days ns; 3 days *vs.* 5 days ns; GelMA_HIGH_7.5: 0 days *vs.* 1 day ns; 1 day *vs.* 3 days ns; 3 days *vs.* 5 days ns; GelMA_HIGH_10 0 days *vs.* 1 day ns; 1 day *vs.* 3 days ns; 3 days *vs.* 5 days ns).

The behavior of GelMA_LOW and GelMA_HIGH gels in contact with fluids was studied by incubating disk-shaped samples in PBS at 37°C. The samples were then collected at different time points and their wet and dried weight values were used to estimate gel swelling ratio after 1, 3 and 5 days of incubation. The initial gel swelling ratio, also known as relaxed swelling ratio (Q_mr_), was also estimated using the wet and dried weights of as-prepared gels. Figure 6 summarizes the swelling ratio values of GelMA_LOW and GelMA_HIGH gels as a function of incubation time in PBS. Focusing on relaxed swelling ratio data, a significant decrease (*p* < 0.001) in this parameter was observed with increasing gel polymeric concentration, in agreement with their increased GelMA content. Accordingly, the degree of methacryloylation did not significantly affect the relaxed swelling ratio data, with the exception of GelMA_LOW_5% and GelMA_HIGH_5% samples that showed significantly different Q_mr_ values (*p* < 0.0001). This discrepancy is likely due to higher variability in the wet and dry weights of these samples, which can be ascribed to their very soft consistency and the consequent difficulty in handling them. Irrespective of their composition, GelMA_LOW-based gels showed higher responsiveness to the surrounding PBS medium, with swelling ratio values increasing over time for all the tested formulations. In particular, GelMA_LOW_5 exhibited the highest responsiveness in contact with fluids and its swelling ratio showed a *ca.* 64% increase upon 1 day of incubation in PBS. This result can be correlated to the different composition of the gels: with increasing GelMA_LOW concentration, samples showed less relevant (although still significant) changes in their swelling ratio in agreement with their denser crosslinked network and smaller pore size (after 1 day of incubation, GelMA_LOW_7.5% and GelMA_LOW_10% increased their swelling ratio by 46% and 30%, respectively). Conversely, GelMA_HIGH-based gels kept their swelling ratio values almost constant over time (no significant differences were observed for all formulations over time), suggesting for these samples the capability to keep the balance between wet and dried weight unaltered and thus a lower responsiveness to surrounding fluids and an enhanced shape retention during incubation. The opposite behavior of GelMA_LOW and GelMA_HIGH gels during incubation in fluids also reflected on their structural properties, as evidenced by Table 2 that reports the estimated mesh size values exhibited by the gels after 1, 3 and 5 days of immersion in PBS at 37°C (slight discrepancies among mesh size data reported in Tables 1, 2 can be ascribed to the different approach used for their estimation, the former being based on rheological data and the latter on sample wet and dried weight). In detail, while GelMA_LOW-based gels exhibited variability in their mesh size, showing a general increasing trend consistent with their progressively increasing swelling ratio over time, GelMA_HIGH-based samples maintained an almost unchanged mesh size throughout the incubation period, further supporting their lower responsiveness to fluid environments and enhanced stability.

**TABLE 2:** Mesh size (ξ) exhibited by GelMA_LOW and GelMA_HIGH photo-crosslinked gels after 1, 3 and 5 days of incubation in PBS at 37°C. Data were estimated from the gel weight measured after incubation in fluids, the volume fraction and the GelMA hydrogel characteristic parameters.

GelMA	Concentration	Mesh size (ξ, nm)		
		1day	3days	5days
GelMA_LOW	5% w/v	20.2	16.9	22.0
	7.5% w/v	10.5	9.4	12.3
	10% w/v	6.4	9.9	7.6
GelMA_HIGH	5% w/v	7.5	7.9	7.5
	7.5% w/v	4.1	4.5	4.2
	10% w/v	3.5	3.0	4.1

### 3.2 hiPSC-CMs differentiation

**FIGURE 7:**
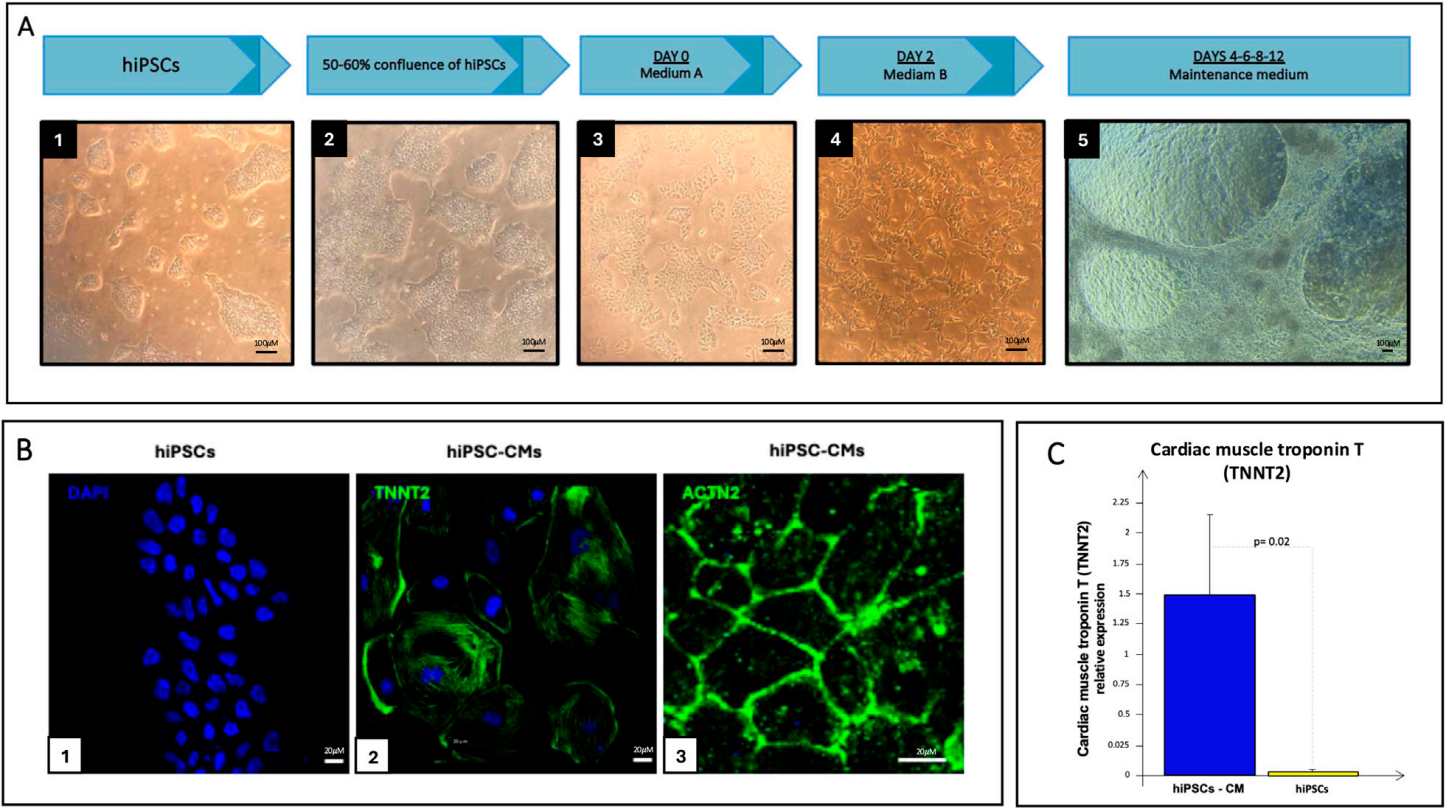
**(A)** hiPSCs differentiation process: starting from undifferentiated clusters of hiPSCs (A1-A4, scale bar 100 *μ*M) to beating hiPSC-CMs (A5, scale bar 100 *μ*M) using a 12-day differentiation protocol; **(B)** Immunofluorescence staining of hiPSCs (B1, scale bar 20 *μ*M) and hiPSC-CMs (B2-B3, scale bar 20 *μ*M): nucleus in blue (DAPI), TNNT2 or ACTN2 in green (Alexa Fluor 488); **(C)** Real-Time PCR of TNNT2 in hiPSCs (yellow bar) and hiPSC-CMs culture (blue bar).

hiPSCs, organized into clusters, were differentiated into beating hiPSC-CMs with a protocol that lasted 12 days (Figure 7A). As evidenced by pictures, cells amplified and changed their shape and organization during differentiation until they covered the entire surface of the flask, and, at some points, different layers of cells overlapped each other. The differentiation of hiPSCs into CMs was confirmed by immunofluorescence staining of the cardiac structural and functional markers cardiac troponin (*TNNT2*) and actinin-α 2 (*ACTN2*), directly involved in the contraction process, which presence was recorded only in the hiPSC-CMs population but not in undifferentiated hiPSCs (Figure 7B). These data were confirmed by Real-Time PCR analysis, which evidenced a higher expression of the *TNNT2* gene in differentiated CMs compared with the hiPSCs population (Figure 7C).

### 3.3 Effect of HCAECs and 3D microenvironment on hiPSC -CMs maturation

**FIGURE 8:**
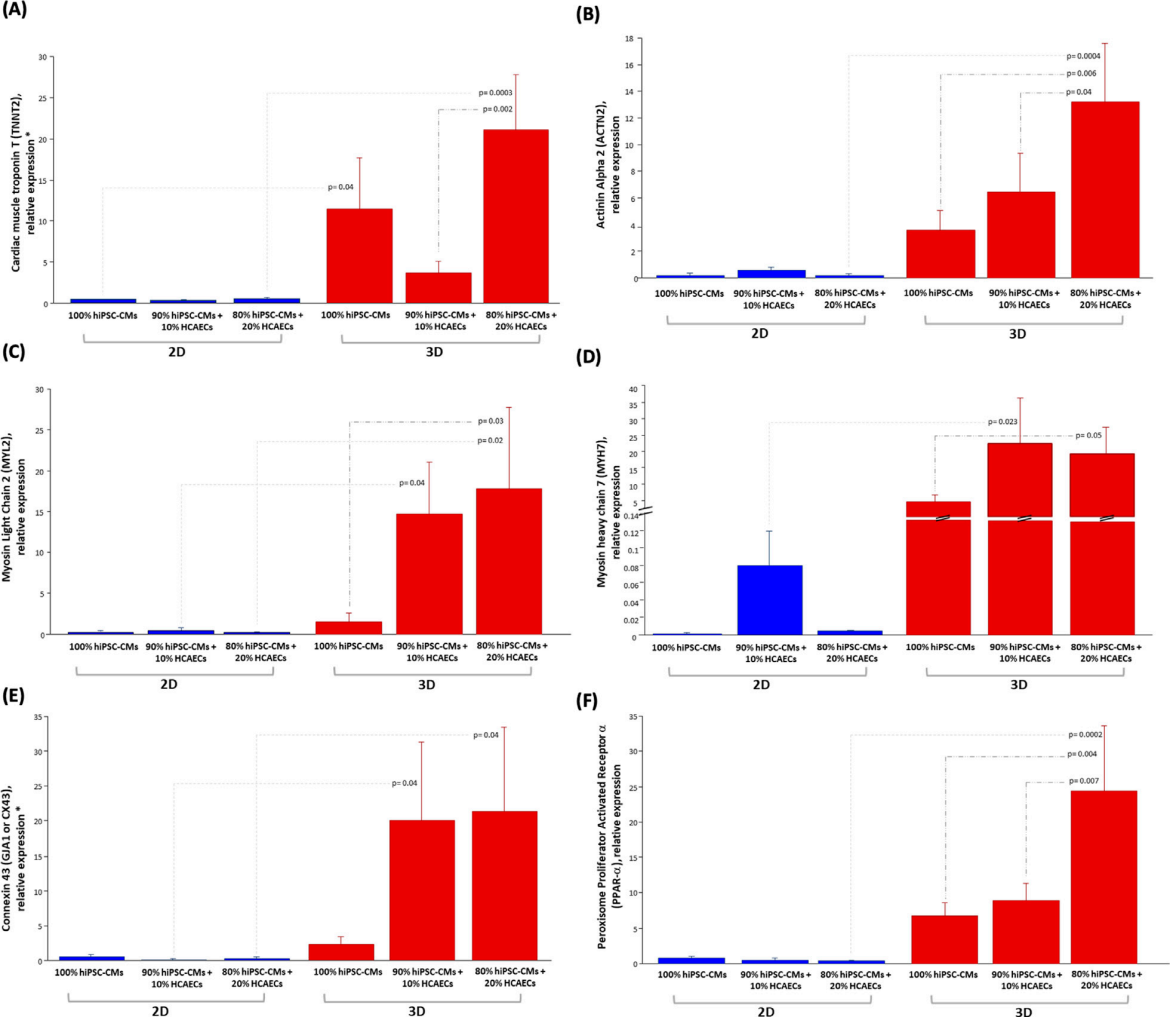
Relative mRNA expression levels of: cardiac maturation structure genes **(A)** TNNT2, **(B)** ACTN2, **(C)** MYL2, **(D)** MYH7; Gap junction gene **(E)** CX43 or GJA1; gene of metabolism **(F)** PPAR-α in hiPSC-CMs monoculture (100% hiPSC- CMs) and co-culture with HCEACs (90% hiPSC-CMs + 10% HCAECs and 80% hiPSC-CMs + 20% HCAECs) in 2D (blue bars) and 3D (red bars) microenvironment. The mRNA expression data were normalized by the geometric mean of the most stably expressed genes (RPL13a, RPS4X, and PPIA), and the relative quantification was performed by the ∆∆Ct method. The Fisher’s test was used after ANOVA and the results were expressed as mean ± SEM (p-value <0.05 was considered significant). (N = 2, n = 3).

To evaluate the impact of both HCAECs and 3D organization on hiPSC-CMs maturation, the expression of several differentiation markers was analyzed by quantitative RT-PCR in both 2D and 3D co-cultures. To this purpose, two different hiPSC-CMs to HCAECs ratios (90% hiPSC-CMs + 10% HCAECs and 80% hiPSC-CMs + 20% HCAECs) were co-cultured for 14 days, in parallel with a monoculture of hiPSC-CMs (100% hiPSC-CMs), simultaneously in a classical 2D system *versus* the 3D microenvironment established using GelMA_HIGH (hereafter referred to with GelMA) hydrogel at 5% w/v concentration containing LAP as photoinitiator (at 0.05 %w/v concentration) and crosslinked by UV light irradiation at 365 nm and 10 mW/cm^2^ for 40 s. At the end of the culture period, specific cardiac maturation markers were analysed. As shown in Figure 8, a significantly higher expression of the cardiac maturation structure markers troponin T2 (*TNNT2*), alpha-actinin 2 (*ACTN2),* ventricular regulatory myosin light chain (*MYL2*) and cardiac myosin heavy chain-7B (*MYH7*), all involved in the cardiac contraction process, was registered in the 80% hiPSC-CMs + 20% HCAECs co-culture established in the 3D environment rather than both the monoculture system and all the 2D cell culture conditions (Figures 8A–D). The gap junction gene (*GJA1/CX43*), encoding for the cardiac connexin 43, also resulted significantly more expressed in the 3D co-culture system (Figure 8E), as well as the key regulator of the lipid metabolism (peroxisome proliferator-activated receptor alpha, *PPAR-α*), typical of mature hiPSC-CMs, which was significantly increased in the 80% hiPSC-CMs + 20% HCAECs 3D co-culture setup (Figure 8F).

The CellTiter-Blue assay evidenced a significantly higher viability for cells co-cultured in the 3D system than a monoculture of hiSPC-CMs (Supplementary Material Figure S2). Moreover, the viability of the 90% hiPSC-CMs + 10% HCAECs and 80% hiPSC-CMs + 20% HCAECs groups in the 3D system was significantly higher than that of the same groups cultured in the 2D microenvironment, demonstrating an improved survival and proliferation condition for cells maintained in this current setup.

### 3.4 Biological characterization of 80% hiPSC-CMs+ 20% HCAECs co-culture in the 3D system *versus* the hiPSC-CMs monoculture in the classical 2D model

**FIGURE 9:**
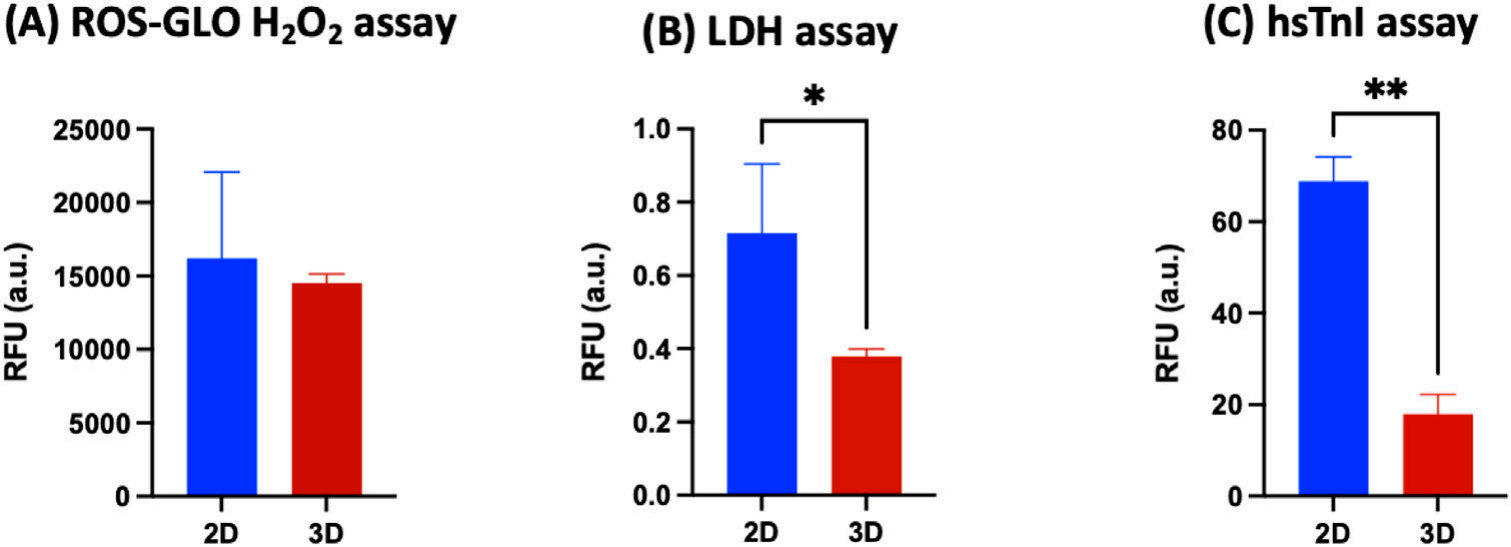
**(A)** ROS-GLO H_2_O_2_ assay, **(B)** LDH assay, and **(C)** high sensitive Troponin I detection immunoassay on hiPSC-CMs monoculture in the 2D system (blue bars) *versus* 80% hiPSC-CMs + 20% HCAECs co-culture condition in the 3D system (red bars) (at least N = 3, n = 3). Parametric t-test was used in **(A,B)**, non-parametric t-test (Mann-Whitney test) was used in **(C)** (*p < 0.05; **p < 0.01).

**FIGURE 10:**
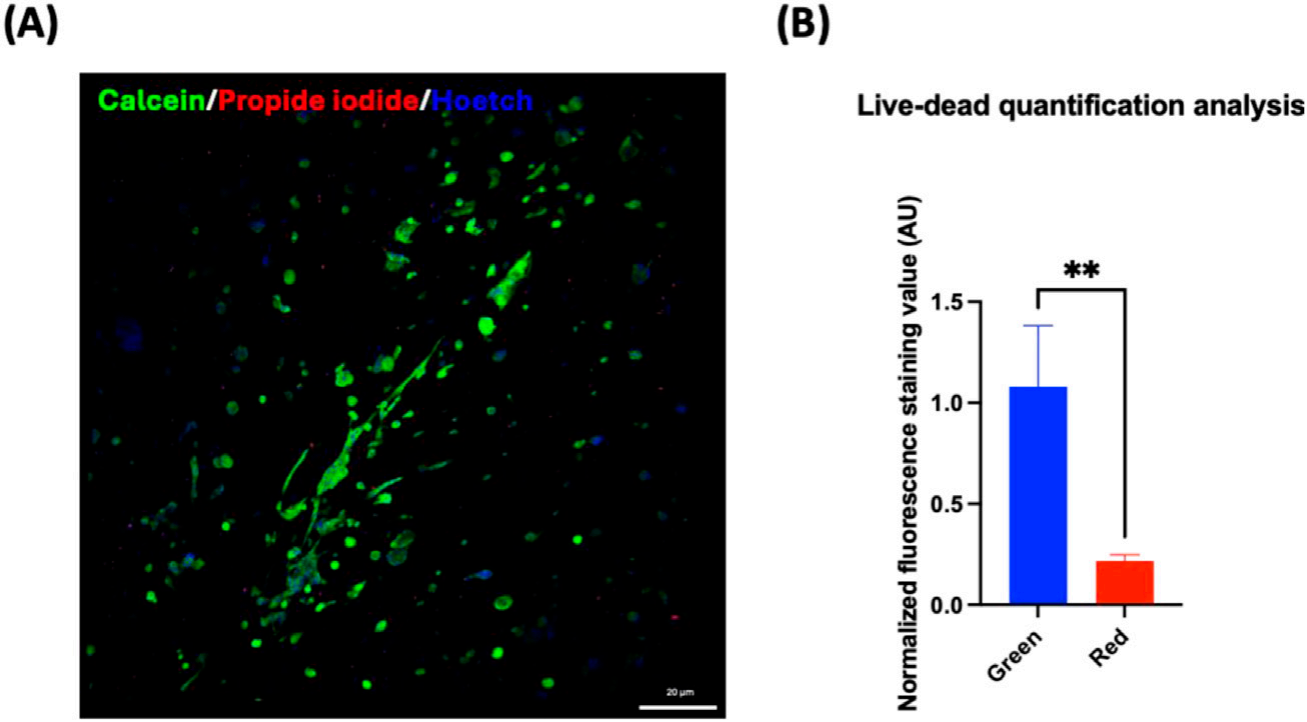
**(A)** Live-dead assay on 80% hiPSC-CMs + 20% HCAECs co-culture in the 3D microenvironment for 14 days. Live cells are stained green (Calcein), dead cells red (Propidium iodide), and nuclei blue (Hoechst). **(B)** Statistical analysis (Unpaired t-test, **p < 0.01) for quantification of green (calcein AM) and red (propidium iodide) fluorescence staining, indicating live and dead cells, respectively. Green and red fluorescence values were normalized to Hoechst fluorescence.

Data obtained from the direct comparison of viability and cardiac maturation markers of hiPSC-CMs cultured alone or with HCAECs in a classical 2D *versus* 3D microenvironment showed a significantly increased degree of maturity achieved in hiPSC-CMs co-cultured with HCAECs (80% hiPSC-CMs + 20% HCAECs condition) in the developed 3D GelMA system. This condition was selected as the best one to focus on in order to study cell health and behavior compared with a standard 2D monoculture of hiPSC-CMs. Some preliminary biological characterization assays (ROS-Glo H_2_O_2_, LDH, and hsTnI assays) were performed at the end of the 14 days of culture. The ROS H_2_O_2_ assay highlighted a reduction trend of stress on cells co-cultured in a 3D environment compared to the classical 2D system (Figure 9A). In parallel, the LDH and hsTnI assays evidenced a significant reduction of release of both LDH enzyme and hsTnI in the 3D microenvironment with respect to the 2D setup, supporting the idea of reduced suffering for cells in the 3D system (Figures 9B,C). Furthermore, the Live-Dead assay performed in a 3D microenvironment confirmed good cell survival after 14 days of co-culture in this configuration, as demonstrated by the significantly increased presence of the green dye Calcein with respect to the almost absent dye Propidium iodide (red staining) (Figure 10).

### 3.5 Transcriptomics and proteomics analysis

Proteomic datasets, obtained from 3D co-culture in GelMA (80% hiPSC-CMs + 20% HCAECs) and monoculture of 100% hiPSC-CMs in the 2D system, were analyzed to assess any differences in gene and protein expression between the two experimental settings. The top 100 differentially expressed genes and proteins are shown in the Supplementary Material Figures S3, S4, respectively. The biological processes (BPs), cellular components (CCs), and molecular functions (MFs) involved in cardiac function and maturation were investigated in more detail by Gene Ontology (GO) analysis. Common BPs, CCs and MFs between 2D and 3D models are reported in Table 3. In detail, GO terms related to cardiac muscle contraction in terms of biological processes (muscle contraction and protein folding), their related cellular components (sarcomere and myofibrils organization, mitochondrial membrane), and molecular functions (actin filament binding, cadherin binding) were identified in both the 2D and the 3D systems, with a higher number of proteins activated (in general terms) in the 3D system when comparing the biological processes and cellular components. This highlights the increased activity related to muscular functions in the cellular machinery of the 3D microenvironment. Similarly, through transcriptomic analysis specific genes were highlighted and quantified in the 2D monoculture (20,418) and 3D co-culture systems (13,699), then analysed and compared through GO. As reported in Table 4, BPs connected to muscle contraction (i.e., regulation of cardiac muscle contraction, regulation of supramolecular fiber organization, sarcomere organization) were registered in both 2D and 3D systems, together with CCs (i.e., cardiac myofibril, actin cytoskeleton, sarcolemma, supramolecular fiber) and MFs (cytoskeletal protein binding, translation regulator activity) associated with them. As observed in proteomics results, a higher number of genes are expressed in 3D compared to 2D systems, evidencing the higher similarity of cells grown in a 3D system to be closer to the natural heart tissue (Stomer, 1998).

**TABLE 3:** Gene ontology (GO) analysis of proteomics data of 2D and 3D models. Common biological processes (BPs), cellular components (CCs), and molecular functions (MFs) based on proteomics data. The number of identified proteins and the False Discovery Rate (FDR) were reported for each term.

Outcomes of GO Analysis	GO term	2D		3D	
		Number of proteins	FDR	Number of proteins	FDR
Biological Processes (BP)	Intracellular transport	42	7.5E-03	50	2.02E-06
	Muscle contraction	17	1.60E-04	17	3.18E-05
	Protein folding	12	3.23E-02	22	3.02E-09
Cellular Components (CC)	Focal adhesion	21	2.50E-04	47	8.14E-24
	Myofibril	20	3.74E-07	17	6.03E-06
	Sarcomere	19	5.06E-07	16	9.29E-06
	Mitochondrial membrane	24	2.46E-02	66	4.06E-28
Molecular Functions (MF)	Actin filament binding	17	4.12E-02	16	4.64E-05
	Cell adhesion molecule binding	70	9.94E-19	45	4.88E-16
	Cadherin binding	59	1.18E-21	32	5.76E-13
	Electron transfer activity	17	1.00E-04	18	1.09E-09
	Oxidoreductase activity	17	1.60E-04	57	7.07E-20

**TABLE 4:** Gene ontology (GO) analysis of transcriptomics data of 2D and 3D models. Common BPs, CCs, and MFs in 2D and 3D environments, based on transcriptomic data. The number of identified genes and False Discovery Rate (FDR) were reported for each term.

Outcomes of GO Analysis	GO term	2D		3D	
		Number of genes	FDR	Number of genes	FDR
Biological Processes (BP)	Regulation of RNA splicing	55	4.51E-10	61	1.13E-11
	Endoplasmic reticulum calcium ion homeostasis	10	2.05E-02	11	7.50E-03
	Muscle filament sliding	6	4.68E-02	6	4.13E-02
	Muscle tissue development	64	8.97E-06	74	7.63E-08
	Regulation of cardiac muscle contraction	17	3.53E-02	19	1.04E-02
	Regulation of cytoskeleton organization	36	7.50E-03	31	1.80E-02
	Regulation of heart contraction	41	5.70E-04	42	8.20E-04
	Regulation of supramolecular fiber organization	64	7.60E-04	94	2.20E-11
	Sarcomere organization	21	4.81E-07	23	6.43E-08
Cellular Components (CC)	Actin cytoskeleton	82	1.29E-05	144	7.88E-25
	Cardiac myofibril	6	2.70E-03	4	4.93E-02
	Cell junction	301	1.25E-11	427	4.79E-39
	Sarcolemma	26	8.30E-03	27	7.40E-03
	Supramolecular fiber	145	6.23E-06	197	6.67E-16
Molecular Functions (MF)	Cadherin binding	92	9.05E-15	146	1.21E-38
	Cytoskeletal protein binding	141	2.60E-04	233	3.20E-26
	Oxidoreductase activity	128	2.17E-08	123	8.63E-06
	Titin binding	8	1.39E-02	8	1.10E-02
	Translation regulator activity	45	8.73E-08	64	1.05E-15

## 4 Discussion

The immature phenotype of cardiomyocytes obtained from hiPSCs (hiPSC-CMs) represents an important limit to consider in *in vitro* cardiovascular disease modeling and toxicological screening studies using these promising cells as an alternative to animals. Several studies have indeed demonstrated that the maturation profile of hiPSC-CMs is closer to the primary fetal instead of the adult ventricular CMs (Denning et al., 2016; Mills and Hudson, 2019; Ahmed et al., 2020; Silbernagel et al., 2020; Camman et al., 2022). Alternatives proposed to overcome immaturity are based on a better reproduction of the *in vivo* CMs microenvironment by co-culturing with different cell types, exposing to specific stimuli, embedding in ECM proteins (Sturtzel, 2017; Lother et al., 2018; Dunn et al., 2019; Giacomelli et al., 2020; Zhao et al., 2020; Campostrini et al., 2021; Ni et al., 2021; Gisone et al., 2022), or replicating a three-dimensional culture to guide cell maturation, getting closer to the organization that occurs in the *in vivo* organism (Ahmed et al., 2020; Camman et al., 2022; Gisone et al., 2022; Zhang et al., 2024). Among the most widely explored biomaterials for cardiac tissue engineering applications, gelatin and its derivatives hold great promise due to their compositional similarity to the native cardiac ECM, biocompatibility, low immunogenicity, biodegradability, viscoelastic properties and low cost. Based on these premises, in the present work, a stimulating environment to enhance hiPSC-CMs maturation was reproduced by combining a 14-days co-culture of hiPSC-CMs and HCAECs in a 3D gel system composed of a custom-synthesized GelMA biomaterial. First, GelMA was synthesized by reacting porcine gelatin type A with methacrylic anhydride and the success of the synthesis process was assessed by ATR-FTIR and ^1^H NMR spectroscopies (Figures 1, 2). While ATR-FTIR spectroscopy did not directly prove GelMA successful synthesis because the characteristic peaks of methacryloyl groups and unmodified gelatin were overlapped, ^1^H NMR spectra provided definitive proof that methacryloyl moieties were effectively grafted to the gelatin backbone. Furthermore, ^1^H NMR spectroscopy also evidenced that GelMA was successfully synthesized with two degrees of methacryloylation by tuning the amount of MA added to the gelatin solution to initiate the bulk functionalization reaction. Indeed, in Figure 2 the peak due to lysine methylene groups decreased with increasing MA content, while the signals produced by the acrylic and methyl protons of methacryloyl groups followed an opposite trend, i.e., the peak intensity increased with increasing the MA amount. The ninhydrin colorimetric assay further supported this result, highlighting that GelMA was effectively synthesized with LOW or HIGH DoM (i.e., between 30%–40% and 96%–97%) by adding MA at 0.1 or 1 mL/g_gelatin_ content, respectively (Figure 3). Overall, good repeatability of the synthesis process was also observed, with lower DoM variability for GelMA_HIGH compared to GelMA_LOW (Figure 3). Indeed, greater synthesis repeatability was clearly visible when the amount of methacrylic anhydride added to the gelatin solution was increased to induce the methacryloylation reaction. Such a trend is most likely due to the progressive negligibility of the side reaction occurring between methacrylic anhydride and water molecules, with increasing the concentration of methacrylic anhydride within the reaction mixture. GelMA_LOW and GelMA_HIGH hydrogels were then prepared at 5, 7.5 and 10% w/v concentrations by solubilizing the material in an aqueous medium containing LAP as photoinitiator. Photo-rheological tests clearly evidenced the light-responsiveness of the developed hydrogels during irradiation at 365 nm and 10 mW/cm^2^ (Figure 4A). Furthermore, these data highlighted the feasibility of finely modulating the mechanical properties of GelMA gels by changing GelMA DoM and/or concentration within the starting hydrogel aqueous solutions, achieving Young’s modulus values within *ca.* 4 and 55 kPa (Figure 5). The estimated Young’s modulus of the designed GelMA gels fell within the stiffness range of biological tissues and cells (E < 100 kPa) (Yuk et al., 2019), which makes them highly promising in soft tissue engineering applications. Rheological data also provided information on the structural properties of GelMA_LOW and GelMA_HIGH gels, evidencing increasing crosslinking densities with increasing polymeric concentration within the starting hydrogel aqueous solutions or with increasing GelMA DoM (Table 1). Accordingly, gel mesh size decreased with increasing crosslinking density and showed values ranging between 7 and 14 nm depending on GelMA DoM and hydrogel polymeric concentration (Table 1). The developed gels were also characterized for their behavior during immersion in fluids under physiological-like conditions (i.e., in PBS at 37°C) showing concentration- and DoM-dependent swelling ratio values (Figure 6). Specifically, irrespective of GelMA DoM, gels prepared at higher polymer concentration showed lower swelling ratio, in agreement with their higher crosslinking density and decreased pore size. Conversely, GelMA DoM turned out to play a pivotal role in determining gel responsiveness to surrounding fluids. In particular, gels prepared starting from GelMA_LOW showed a significantly higher responsiveness to surrounding fluids, while similar formulations prepared using GelMA_HIGH kept their swelling ratio almost constant over time. This result suggested that GelMA_HIGH based samples were able to keep the balance between wet and dried weight unaltered over time, showing a lower responsiveness to surrounding fluids and enhanced shape retention during incubation.

Photo-cured gels prepared starting from GelMA_HIGH aqueous solutions at 5% w/v concentration showed an E value (8.70 ± 0.12 kPa) similar to the native cardiac tissue, which Young’s modulus has been reported to range between 8 and 15 kPa (Emig et al., 2021). Furthermore, this formulation showed a mesh size of *ca.* 11 nm, that made it suitable for nutrients and cell metabolites exchange. Indeed, this mesh size is higher than that of nutrients (glucose and NaCl have a Stokes radius of 3.8 and 1.4 Å, respectively (Bhattacharya et al., 2012)) and metabolites that are small molecules with molecular weight typically lower than 1.5 kDa. Lastly, considering the behavior of the gels in aqueous environments, GelMA_HIGH_5% hydrogels demonstrated prolonged stability over time, with the swelling ratio remaining nearly constant at approximately 20 for up to 5 days of incubation. This observed stability indicates the potential of these gels to offer sustained mechanical and structural support to the developing tissue. Based on these premises, GelMA_HIGH_5% was selected to design a 3D environment providing cells with biochemical and mechanical stimuli replicating the native cardiac tissue microenvironment, being gelatin a derivative of collagen (collagen is the main protein constituent of the cardiac ECM) (LeBar et al., 2021) and the estimated gel Young’s modulus in the typical range of native cardiac tissue (Emig et al., 2021). hiPSC-CMs were differentiated into CMs using a 12-day differentiation protocol, with beating contractions starting approximately at the end. The effective differentiation of stem cells into CMs was confirmed through immunofluorescence staining of cardiac structural and functional markers *TNNT2* and *ACTN2* (Figure 7B), which are directly involved in the contraction process. These markers were observed only in the hiPSC-CMs population, as confirmed through Real-time PCR (RT-PCR), which highlighted the expression of *TNNT2* only in differentiated CMs (Figure 7C). To evaluate the impact of endothelial cells, and the 3D structural cue on hiPSC-CMs maturation, two different hiPSC-CMs to HCAECs ratios (90% hiPSC-CMs + 10% HCAECs and 80% hiPSC-CMs + 20% HCAECs) were co-cultured for 2 weeks and compared to a monoculture of hiPSC-CMs (100% hiPSC-CMs), in the 3D hydrogel *versus* a gold-standard 2D culture. During the first week of culture, cells in the 3D microenvironment stretched and modified their morphology within the GelMA gel, while during the second week, beating contractions started. At the end of the culture period, analysis of specific cardiac maturation and structural gene markers highlighted an effective contribution of both endothelial cells and the 3D environment towards hiPSC-CMs maturation (Figure 8). *TNNT2* and *ACTN2* genes, involved in sarcomere assembly, development, and organization (Giacomelli et al., 2020; Guo and Pu, 2020), showed a significant increase in the 3D co-culture setup compared to the 2D environment, revealing a better organized, functional, and mature contractile structure unit (Figure 8A–B). This result was confirmed by the sarcomere proteins switching from fetal to adult isoform, as evidenced by the higher expression of the adult isoform of the cardiac myosin heavy chain (*MYH7*) and the cardiac myosin light chain (*MYL2*), which also indicated a greater cells differentiation toward a ventricular-like phenotype (Figures 8C,D) (Guo and Pu, 2020; Singh et al., 2023). Specifically, *TNNT2, MYL2*, and *MYH7* genes contribute to sarcomere formation during differentiation. As cells achieve a more mature profile, the expansion of myofibrils is associated with the formation of additional sarcomeres, resulting in a higher expression of the genes mentioned above (Miller et al., 2021). These data revealed a better organized, functional, and mature contractile structure unit in hiPSC-CMs in the 3D co-culture setting. Promising data were also obtained regarding the expression of the cardiac gap junction protein (*GJA1/CX43*), which showed higher expression in the 3D co-culture, suggesting better intercellular interaction and communication relevant to guarantee the synchronous contraction of CMs (Figure 8E). Data reported in the literature demonstrated the role of the CX43 gap junction in the mechanism that guides the maturation (Giacomelli et al., 2020) of hiPSC-CMs. Regarding the metabolism of cells, good results were reported for the 3D co-culture model with respect to the classical 2D system. The switch of the metabolism from glycolysis to fatty acid is an important key element in showing the transition of CMs from fetal to adult phenotype (Mills and Hudson, 2019; Vučković et al., 2022). The significantly higher expression of the key regulator of lipid metabolism *PPAR-α* confirmed the maturation trend of hiPSC-CMs during co-culture with HCAECs within the 3D gel system compared to the monoculture condition and the 2D microenvironment (Figure 8F). Taken together, the analysis of specific cardiac maturation and structural gene markers highlighted an effective contribution of endothelial cells and the 3D environment towards hiPSC-CMs maturation. Furthermore, results from the 3D setting showed a greater cell viability than the classical 2D one, with the co-culture condition having an improved survival and proliferation of cells, as highlighted by the higher viability of cells yielded in this setup compared to hiPSC-CMs monoculture. This result further suggested that the 3D microenvironment helped in reproducing the complex tissue architecture and the consequent cellular crosstalk and interactions pivotal for the survival and maturation of cells. This is in line with the study of Colliva et al., which evidenced the importance of continued paracrine communication between cardiomyocytes and endothelial cells. On the one hand, HCAECs are also important in promoting CMs’ organization, function, and survival. At the same time, CMs produce factors involved in endothelial cell function, creating a beneficial loop for both cell populations (Colliva et al., 2020). After confirming a better maturation profile obtained in the 3D 80% hiPSC-CMs + 20% HCAECs co-culture condition, a deeper biological characterization was carried out for this model, in parallel with a 2D gold-standard monoculture of hiPSC-CMs (Figure 9). The Reactive Oxygen Species (ROS-Glo) production assay, the Lactate dehydrogenase (LDH) assay, and the high sensitive cardiac Troponin I (hsTnI) immunoassay were performed to investigate cell health and behavior, respectively, in terms of oxidative stress and lack of cellular integrity. Cells co-cultured in the 3D system showed a trend of reduced oxidative stress in association with a significantly lower release of LDH enzyme and cTnI, indicating a less critical condition for cell survival and function in the 3D system with HCAECs with respect to the 2D hiPSC-CMs monoculture. These data suggest that the improved viability registered for cells co-cultured in the 3D microenvironment (see Supplementary Material Figure S2) results from higher survival, which is related to a better biological condition for cells, with less stress and suffering. This was also confirmed through the Live-Dead assay performed on cells in the 3D microenvironment, reporting a high number of viable cells after 14 days of co-culture (Figure 10). Indeed, reproducing a microenvironment surrounding CMs similar to the *in vivo* one significantly impacts cells in terms of survival, development, function, and maturation. The comparison between 2D and 3D models was also characterized through the support of Omics. The Gene Ontology (GO) analysis highlighted some common terms in proteomics related to muscle contraction. In detail, proteins related to the sarcomere and myofibrils structures, responsible for heartbeat, were identified in both the 2D and the 3D systems. Biological processes such as muscle contraction or protein synthesis and folding and molecular functions such as actin filament binding and cadherin binding confirmed the synthesis of proteins involved in cardiac muscle contraction (Table 3). Specifically, a higher number of proteins resulted activated (in general terms) in the 3D system when comparing the biological processes and cellular components, thus highlighting the increased activity in the cellular machinery related to muscular functions in the 3D setup. Similarly, the GO analysis performed on transcriptomic data (Table 4) aligned with that obtained from proteomics. Numerous BPs resulted connected to muscle contraction (i.e., regulation of cardiac muscle contraction, regulation of supramolecular fiber organization, sarcomere organization), as confirmed by the presence of many CCs associated with them (i.e., cardiac myofibril, actin cytoskeleton, sarcolemma, supramolecular fiber), with a general higher expression in the 3D model compared to the 2D one. Moreover, transcriptomics results also showed other BPs, CCs and MFs related to the cytoskeleton organization, a key feature for coordinating the sarcomere and the contractile function (Stomer, 1998). These results are in line with the work of Branco et al. (2019), who compared GO analysis of genes from day 15 of hiPSC-CMs differentiated in 2D *versus* 3D aggregates microenvironment, highlighting the upregulation of heart contraction, action potential, signal conduction and cardiovascular system development in the 3D system (Branco et al., 2019). Taken together, these data suggested that 3D organization, in combination with the presence of HCAECs, has a critical impact on the structure and function of hiPSC-CMs that resulted in an effect on the maturation process at the cellular and molecular levels, as demonstrated by the transcriptomics and proteomics analyses, which confirmed and enriched results obtained from the investigation of specific cardiac gene markers expression.

## 5 Conclusion

This work reported the design of an easy-to-assemble 3D *in vitro* bioengineered cardiac tissue model relying on the combination of HCAECs and hiPSC-CMs with gelatin methacryloyl gels. The hiPSC-CMs to HCAECs ratio and the selected GelMA gel composition resulted in a reliable bioengineered replica of the native cardiac tissue, showing improved maturation of CMs towards an adult phenotype within a 3D milieu providing biochemical and mechanical cues similar to the *in vivo* cardiac ECM. Thanks to the integration of bioengineering approaches and biological and omics characterization, this work shows a new way to evaluate the phenotypic changes in different environments, providing new insights into the modulation of cell behavior. Overall, the preliminary data assessed our 3D system as a good model for the proliferation and maturation of healthy beating hiPSC-CMs, opening the way towards further validation of the designed cardiac tissue model given its application in high throughput testing of chemicals (e.g., for assessing cardiotoxicity induced by drugs, stabilizers, and pollutants) and investigating cardiac physiological pathways.

## Data Availability

Data for this article are available upon publication at Zenodo. The biological characterization can be found here: https://doi.org/10.5281/zenodo.13292106. The physico-chemical characterization is available at the following link: https://doi.org/10.5281/zenodo.13271733.
