# Supplementary material for: Integration of co-culture conditions and 3D gelatin methacryloyl hydrogels to improve human-induced pluripotent stem cells-derived cardiomyocytes maturation
Source: Front Bioeng Biotechnol. 2025 Jul 14;13:1576824. doi: 10.3389/fbioe.2025.1576824 (PMC12301886; doi:10.3389/fbioe.2025.1576824)
Supplement: Supplementary file 1 [file DataSheet1.docx]

Supplementary Material

# Supplementary Figures


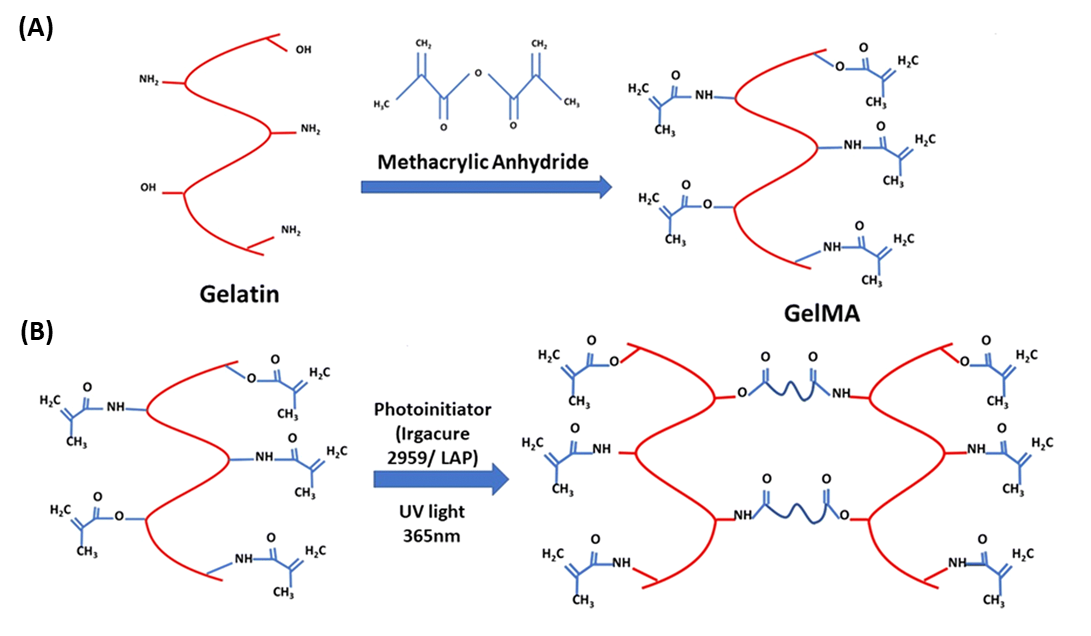


Supplementary Figure S1. Schematic representation of GelMA synthesis (A) and cross-linking through light irradiation in the presence of a photoinitiator (B). Reprinted from Ghosh et al. (Gosh et al. 2023)


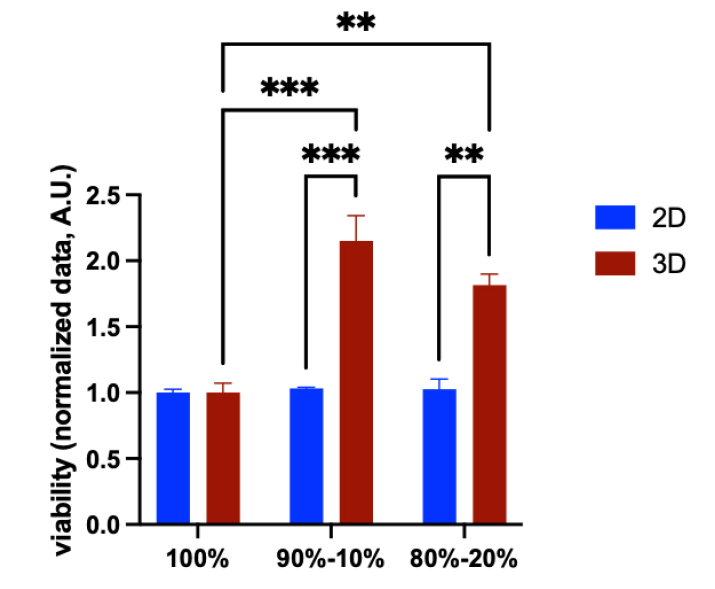


Supplementary Figure S2. Impact of HCAECs and 3D microenvironment on cell viability. CellTiter-Blue® assay of hiPSC-CMs monoculture (100% hiPSC-CMs) and co-culture with HCAECs (90% hiPSC-CMs + 10% HCAECs and 80% hiPSC-CMs + 20% HCAECs) in 2D (blue bars) *versus* 3D microenvironment (red bars) (N=2). 2-way ANOVA (post-hoc Tukey correction) (** p< 0.01; *** p< 0.001).


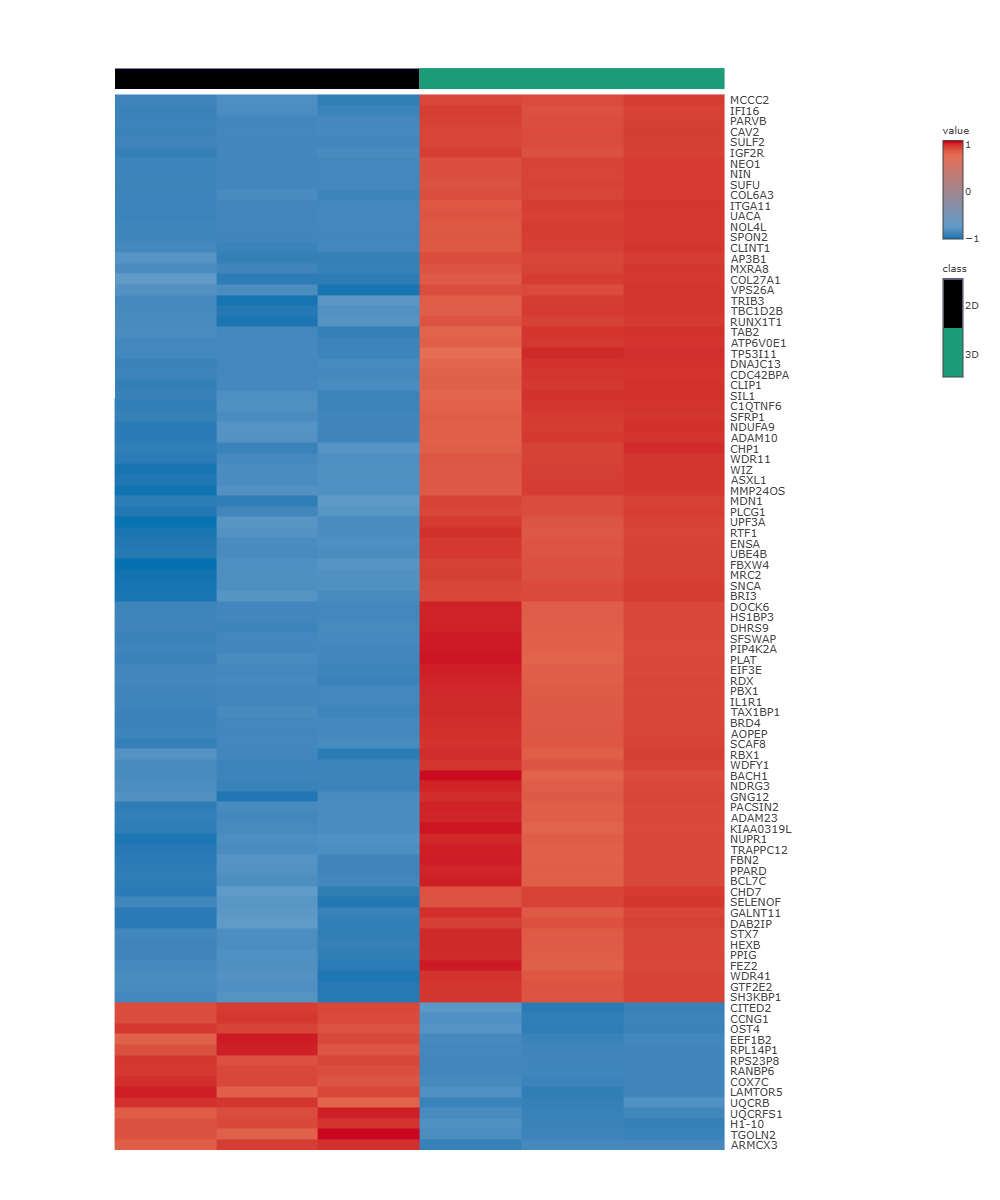


**Supplementary Figure S3.** Heatmap of the top 100 differentially expressed gene profiles of 2D hiPSC-CMs monoculture (100% hiPSC-CMs) and 3D co-culture with HCAECs (80% hiPSC-CMs + 20% HCAECs). Each column represents a biological replicate, and each row represents a gene. The red color indicates gene upregulation, while the blue color indicates downregulation.


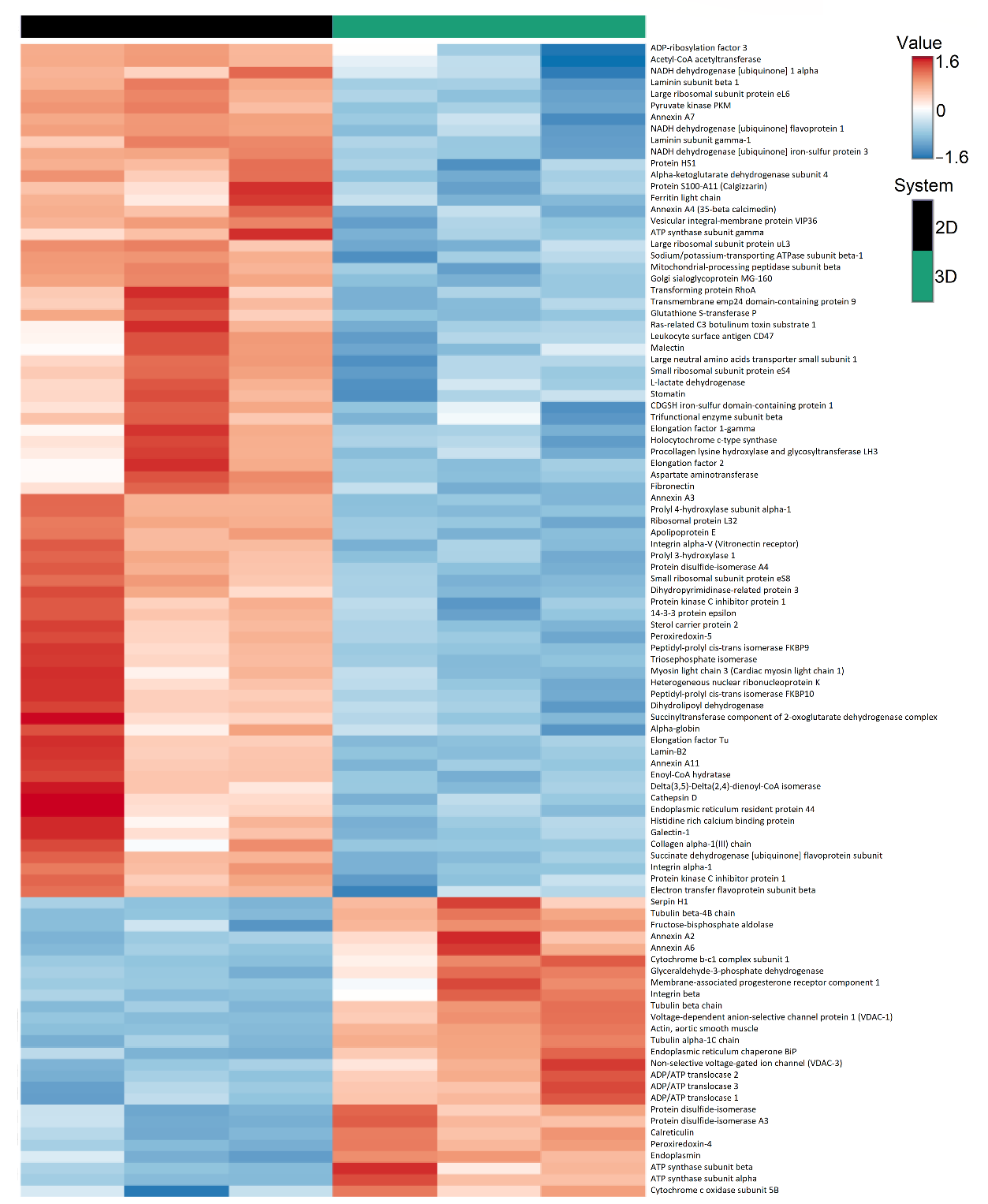


**Supplementary Figure S4.** Heatmap of the top 100 differentially expressed protein profiles of 2D hiPSC-CMs monoculture (100% hiPSC-CMs) and 3D co-culture with HCAECs (80% hiPSC-CMs + 20% HCAECs). Each column represents a biological replicate, and each row represents a protein. The red color indicates protein upregulation, while the blue color indicates downregulation.

# Supplementary Tables


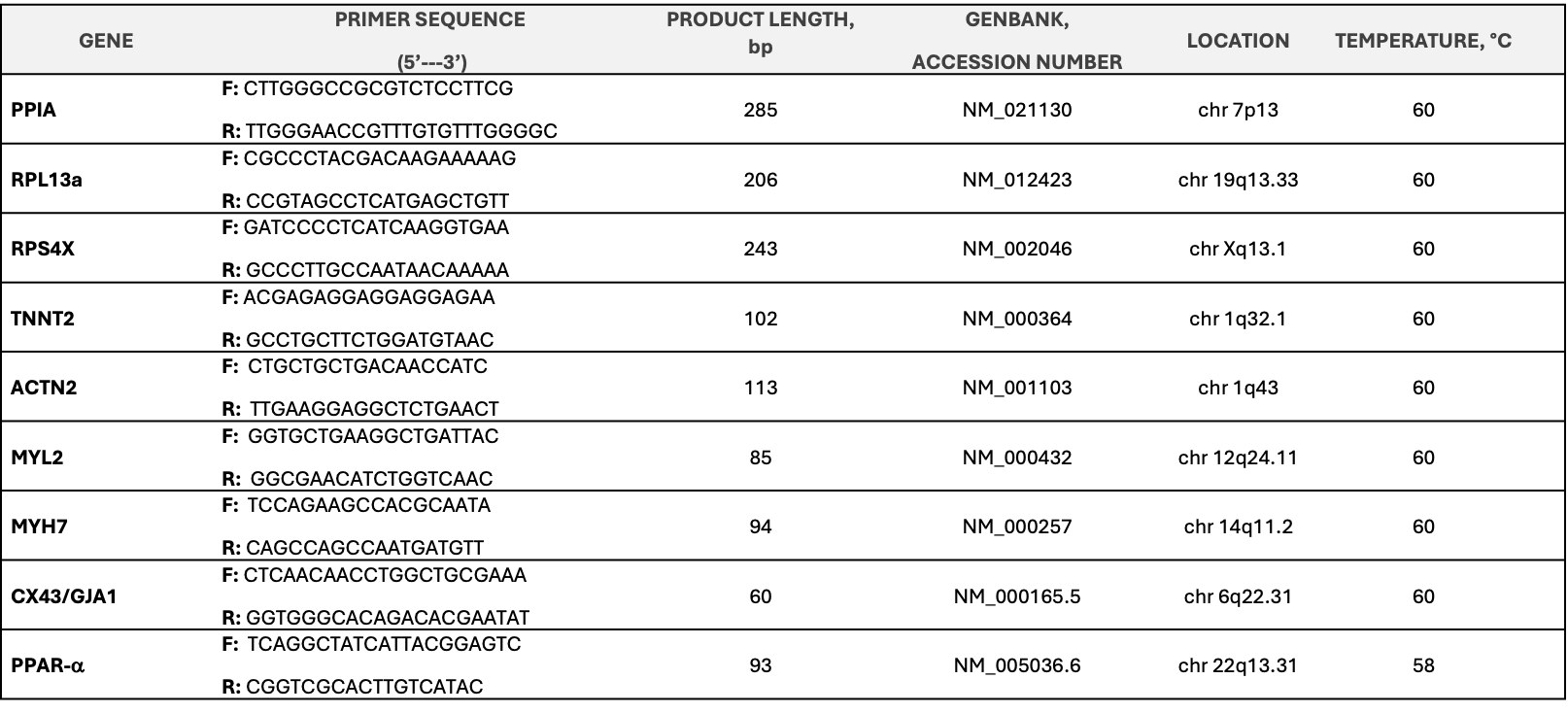


Supplementary table S1. List of primers used for the quantitative RT-PCR analysis. *PPIA*: Peptidylpropyl isomerase A [cyclophillin A]; *RPL13a*: ribosomal protein L13a; *RPS4X*: ribosomal protein S4 X-linked; *TNNT2*: troponin T2, cardiac type; *ACTN2*: actinin alpha 2; *MYL2*: myosin light chain 2; *MYH7*: myosin heavy chain 7; *CX43/GJA1*: connexin 43 or gap junction protein, alpha 1; *PPAR-α:* peroxisome proliferator-activated receptor alpha.

# Supplementary Materials and Methods

**3.1 Evaluation of the mesh size of GelMA gels during incubation in physiological-like conditions**

The mesh size of GelMA gels was calculated from the gel weight measured after incubation in physiological-like conditions (i.e., pH 7.4, 37 °C), the volume fraction and the GelMA hydrogel characteristic parameters, according to the methodology reported by Vigata et al. (Vigata et al., 2021). Briefly, the equilibrium swelling ratio (Q_m_) and relaxed swelling ratio (Q_mr_) were calculated according to Equation 5 reported in the main draft, by referring to the wet and dried GelMA gel weights measured after incubation for a defined time interval (1, 3, and 5 days) and immediately after photo-crosslinking, respectively. The Q_m_ and Q_mr_ values were used to estimate the equilibrium volumetric swelling Q_v_ and the relaxed volumetric swelling Q_vr_, respectively, according to Equation S1

$Q_{v(r)}=1+\frac{\rho_{p}}{\rho_{s}}\left( Q_{m(r)}-1 \right)$ (Eq. S1)

**Equation S1.** Formula used to calculate the equilibrium and relaxed volumetric swelling Q_v_ and Q_vr_

where ρ_p_ (1.35 g/cm^3^) and ρ_s_ (1.014 g/cm^3^) are the polymer density (gelatin) and the solvent density (PBS), respectively.

The equilibrium and relaxed polymer volume fraction (v_2s_ and v_2r_, respectively) were calculated as follows (Equation S2)

$v=\frac{1}{Q_{v}}$ (Eq. S2)

**Equation S2.** Formula used to estimate the equilibrium and relaxed polymer volume fraction

Then, the molecular weight between crosslinks (M_c_, g/mol) was defined by applying the Flory-Rehner equation (Equation S3)

$\frac{1}{M_{c}}=\frac{2}{M_{n}}-\frac{\frac{\bar{\nu}}{V_{1}}\left[ ln\left( 1-v_{2s} \right)+v_{2s}+X_{1}{v_{2s}}^{2} \right]}{v_{2r}\left[ \left( \frac{v_{2s}}{v_{2r}} \right)^{\frac{1}{3}}-\frac{1}{2}\left( \frac{v_{2s}}{v_{2r}} \right) \right]}$ (Eq. S3)

**Equation S3.** Formula used for the definition of the molecular weight between crosslinks

where M_n_ (63,565 g/mol) is the polymer (gelatin) number average molecular weight before crosslinking, $\bar{\nu}$ (0.7407 mL/g) is the specific volume of the polymer, V_1_ (18.01 mL/mol) is the molar volume of water as solvent and X_1_ (0.497) is a parameter expressing the polymer-solvent interaction.

Lastly, the mesh size (ξ, nm) was calculated according to Equation S4

$\xi={v_{2s}}^{-\frac{1}{3}}\times l\left( 2\frac{M_{c}}{M_{r}}C_{n} \right)^{\frac{1}{2}}$ (Eq. S4)

**Equation S4.** Formula used for the estimation of the mesh size of GelMA gels

where M_r_ (91.19 g/mol) is the molecular weight of the repeat unit, l (4.28 Å) is the amino acid bond length, and C_n_ (8.8785) is the Flory’s characteristic ratio for GelMA.

# References

Ghosh R. N., Thomas J., B.R. Vaidehi, N.G. Devi, Janardanan, A., Namboothiri, P. K., et al. (2023). An insight into synthesis, properties and applications of gelatin methacryloyl hydrogel for 3D bioprinting. Mater. Adv. 4, 5496–5529. doi:10.1039/D3MA00715D

Vigata, M., Meinert, C., Bock, N., Dargaville, B.L., Hutmacher, D.W. (2021). Deciphering the Molecular Mechanism of Water Interaction with Gelatin Methacryloyl Hydrogels: Role of Ionic Strength, pH, Drug Loading and Hydrogel Network Characteristics. *Biomedicines* 9, 574. doi: 10.3390/biomedicines9050574
